# Supplementary material for: Robotic platform for microinjection into single cells in brain tissue
Source: EMBO Rep. 2019 Aug 30;20(10):e47880. doi: 10.15252/embr.201947880 (PMC6776899; doi:10.15252/embr.201947880)
Supplement: Supplementary file 1 — Appendix [file EMBR-20-e47880-s001.pdf]

Appendix

Appendix Figure S1 2
Appendix Figure S2 4
Appendix Figure S3 5
Appendix Figure S4 6
Appendix Figure S5 7
Appendix Figure S6....................8
Appendix Figure S7 9
Appendix Figure S8 10
Movie Captions 11
Appendix Note S1: Calibration Procedure 12
Appendix Note S2: Trajectory Generation 14
Appendix Note S3: Calibration consistency across users 15
Appendix Table S1: Parts and Reagents....................16
References 20

Appendix Figure S1

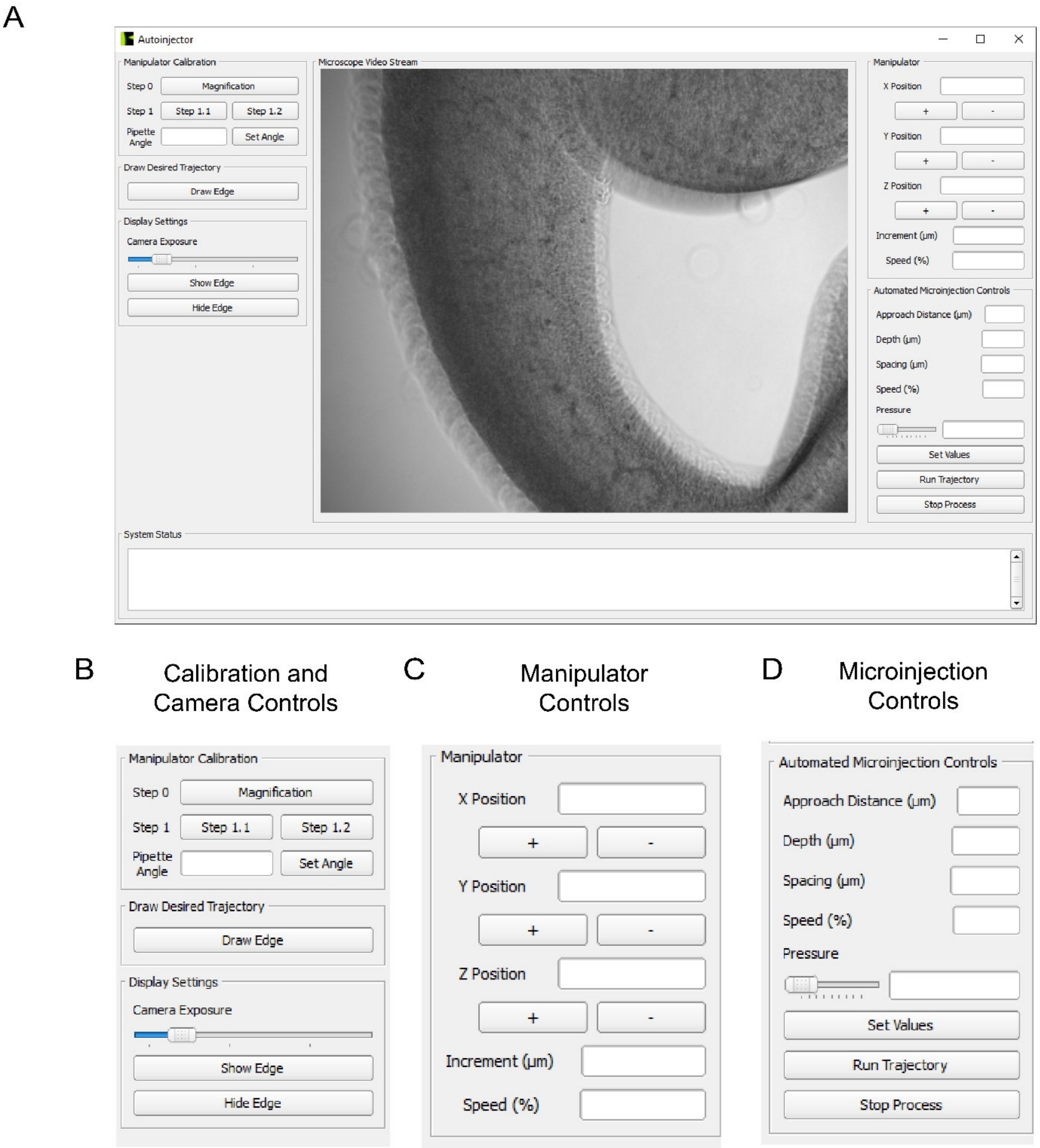

Appendix Figure S1: Autoinjector software graphical user interface (GUI). The GUI developed in Python controls the Autoinjector hardware and allows the user to customize the microinjection protocol. (A) Screenshot of the GUI. Images acquired by the microscope camera are displayed in real time at the center of the GUI. The controls of the microinjection are on the left and right columns of the image. The status of the Autoinjector is displayed in the bottom of the GUI in the response monitor. (B) Controls on the left side of GUI. 'Calibration Controls' are used by the user to enable software control of the manipulator in 3D space using the 2D microscope camera images (see **Appendix Figure. S2A** and **Appendix Note S1** for additional details). The 'Manual Feature Selection' controls allows the user to

annotate the microscope camera images to define the desired path of microinjection. ‘Single Injection Controls’ allow the user to perform a single injection at a given pressure with a given pulse duration. The ‘Video Capture’ controls allow the user to record the real time microscope camera images as a video. The ‘Feature Viewing’ controls allow the user to display or remove the annotated path of microinjection on the microscope camera image feed. (C) Controls on the right side of GUI. ‘Manipulator Controls’ allow the user to see the current position of the manipulator, and to manually advance the manipulator in X, Y, and Z. The ‘Autoinjector Controls’ allow the user to adjust the microinjection parameters such as micropipette compensation pressure, approach distance, depth of injection, spacing between injections, speed of injection, and number of injection attempts (see see **Appendix Figure. S2B** and **Appendix Note S2** for additional details). The ‘Autoinjector Controls’ also allows the user to start the Autoinjector and to stop the process at any moment.

Appendix Figure S2

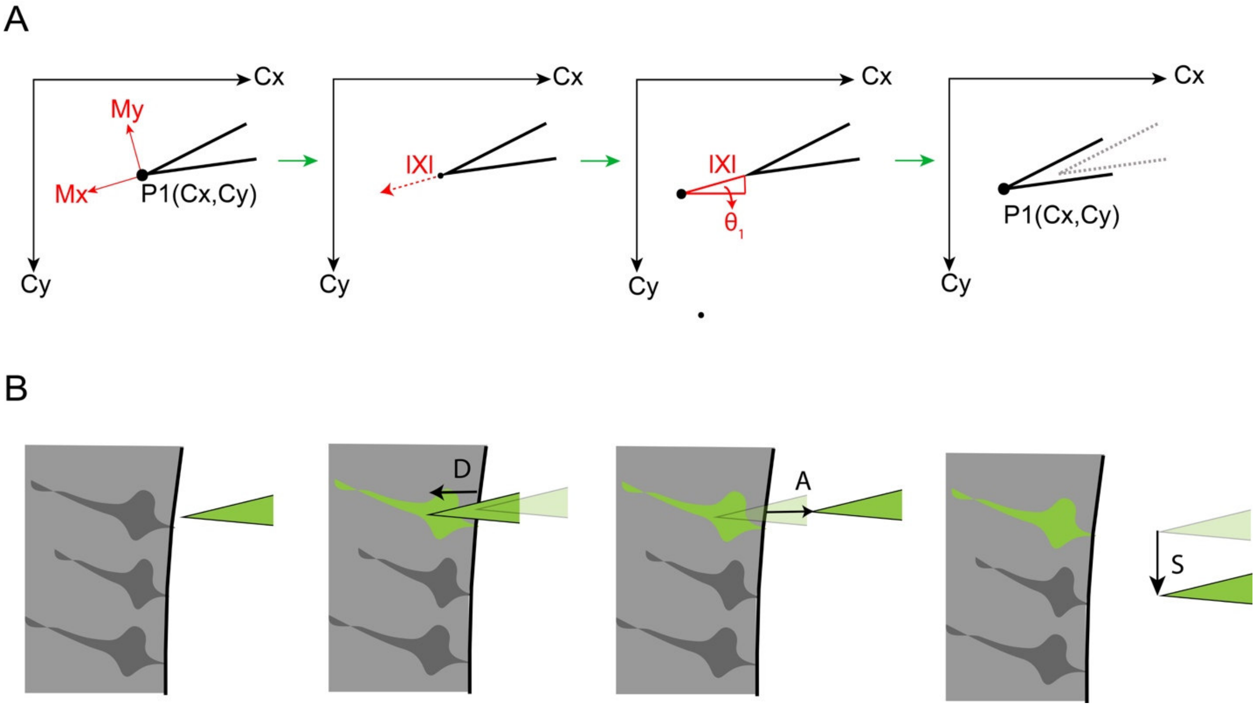

Appendix Figure S2: Autoinjector micropipette manipulator calibration and injection parameter definition. (A) Image guided control of the micropipette required movement in the microscope field of view (FOV) to be translated to the manipulator coordinate system. The micropipette was positioned using the manipulator and had a coordinate system defined by (Mx, My). The tip of the micropipette moved in a second coordinate frame visualized through the microscope FOV defined by (Cx, Cy). The initial position of the micropipette tip in (Cx, Cy) was registered as point P1. The manipulator advanced the micropipette in the manipulator X-axis (Mx) by a predefined distance IXI. The final position of the micropipette tip in (Cx,Cy) was registered as point P2. The distance between P1 and P2 allowed transformation of the displacement IXI to (Cx,Cy) and was used to calculate the angular offset θ between the two coordinate systems. This then allowed programmatically guidance of the micropipette tip to defined points in the camera FOV. (B) The schematic shows the parameters defined by the user to create the final trajectory of the Autoinjector. The micropipette entered a depth, denoted D, into the tissue to perform the microinjection. Next, the micropipette was pulled out a distance termed the approach distance, denoted A. The micropipette was then moved to the next site of injection by moving along the tissue by a distance termed spacing, denoted S. This process was repeated N times where N was the number of injection attempts.

Appendix Figure S3

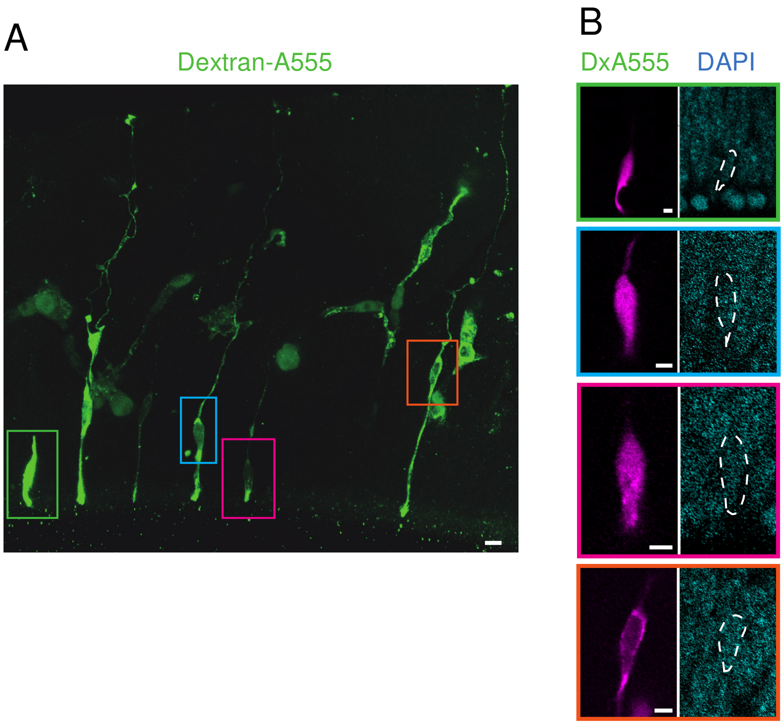

Appendix Figure S3: DAPI staining of microinjected cells. (A) Microinjected cells are as in Figure 3F. Scale bar is 10 µm. (B) High magnification of microinjected cells shown in A and highlighted with a color box. Note the color-coding. Scale bar is 10µm.

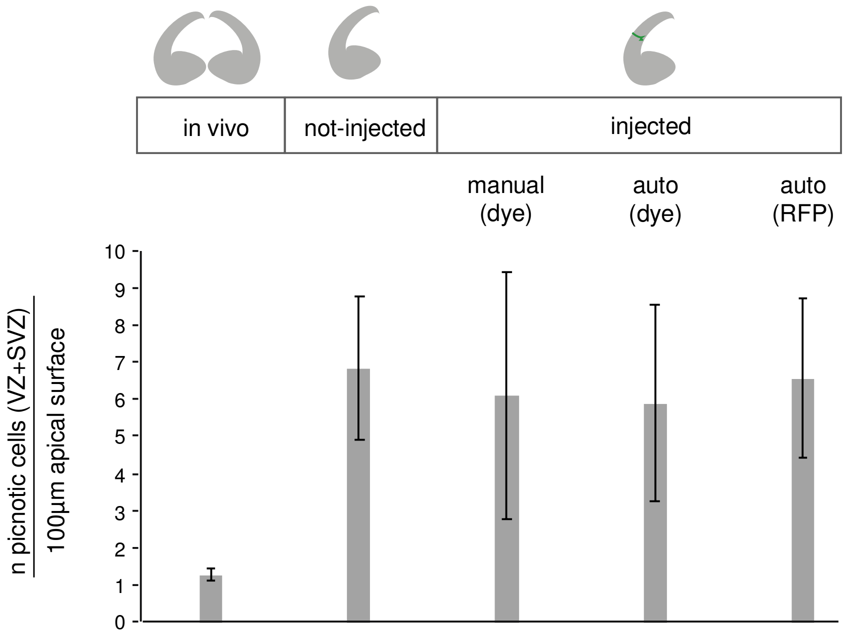

Appendix Figure S4

Appendix Figure 4: Effect of manual and automated injection on tissue viability: picnotic nuclei. Organotypic slices of mouse E14.5 dorsal telencephalon were either (i) non injected (not-injected) (ii) subjected to manual or automated microinjection (injected) using Dextran-A555 (manual and auto, dye) or Dx-A488 along with mRNA for RFP (auto, RFP). Slices were kept in culture for 24h, fixed and stained for DAPI. In order to compare the slice culture system with in vivo situation, E15.5 mouse telencephalon was fixed and stained with DAPI (in vivo). Picnotic nuclei in the (VZ+SVZ) were identified by their DAPI reactivity pattern. The graph (bottom) shows the number of picnotic nuclei per 100µm of apical surface. Note that the slice culture procedure, as expected, increases the number of picnotic nuclei. However, manual and automated microinjection do not increase cell death compared to slice culture alone. In addition, the injection of RFP along with the microinjection dye does not increase cell death.

Appendix Figure S5

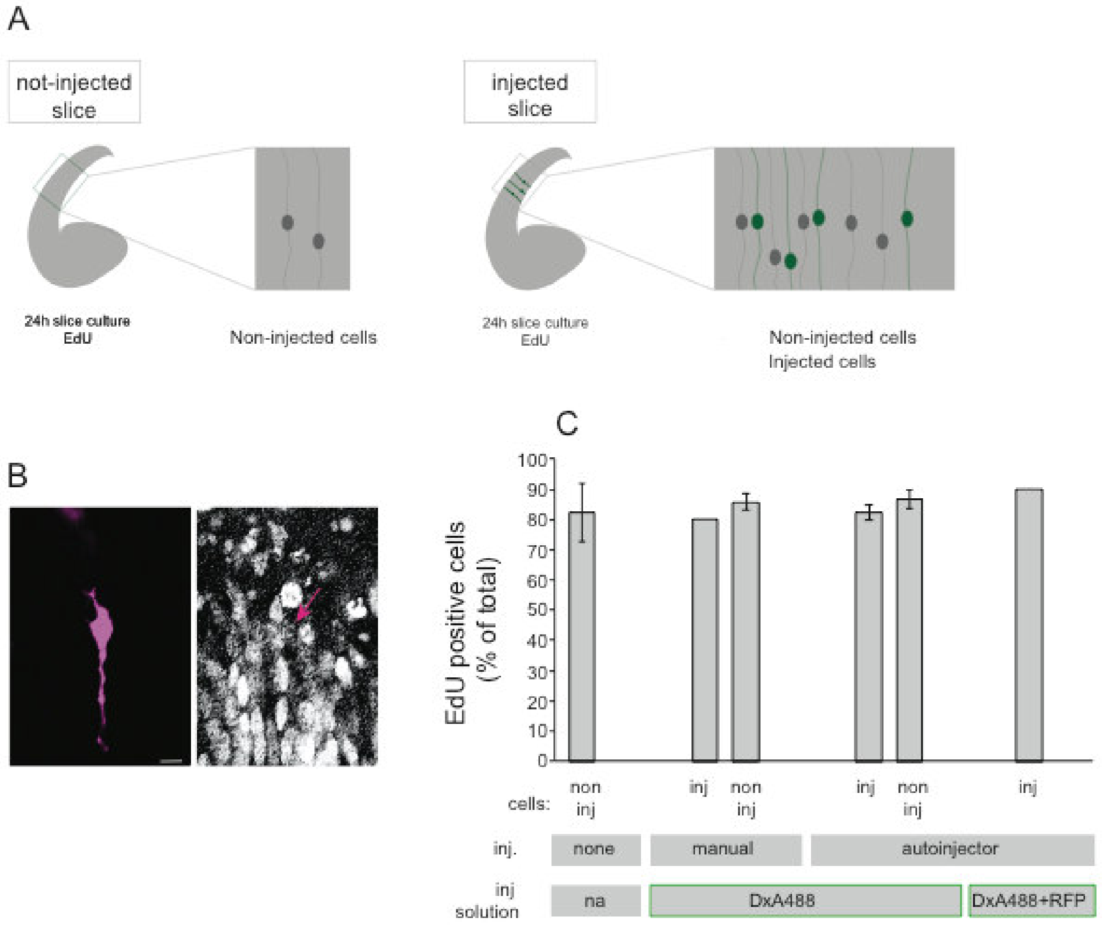

Appendix Figure 5: Effect of manual and automated injection on tissue viability: EdU incorporation. EdU incorporation in microinjected cells and in non-injected cells in the injected area. (A) Schematic of the EdU incorporation experiment. Organotypic slices of mouse E14.5 dorsal telencephalon were either (i) non injected (not-injected slice) or (ii) subjected to manual or automated microinjection (injected slice) using Dextran-A488 (for manual and autoinjector) or Dx-A488 along with mRNA for RFP (autoinjector). Slices were kept in culture for 24h in the presence of EdU, fixed and stained for DAPI and EdU. Both injected cells and non-injected cells in the injected area were scored for their positivity for EdU. (B) Image of EdU incorporation an injected slice (DxA555: magenta; EdU: white). The image is a single optical section, 0.8µm-thick. Scale bar: 10µm. (C) Quantification of EdU positive cells, expressed ad % of total in different experimental conditions. Note that the EdU incorporation pattern is comparable in non-injected slices, injected cells and non-injected cells in an injected area, irrespective of the solution injected (DxA488 or DxA488+RFP) and of the use of manual or automated injection.

Appendix Figure S6

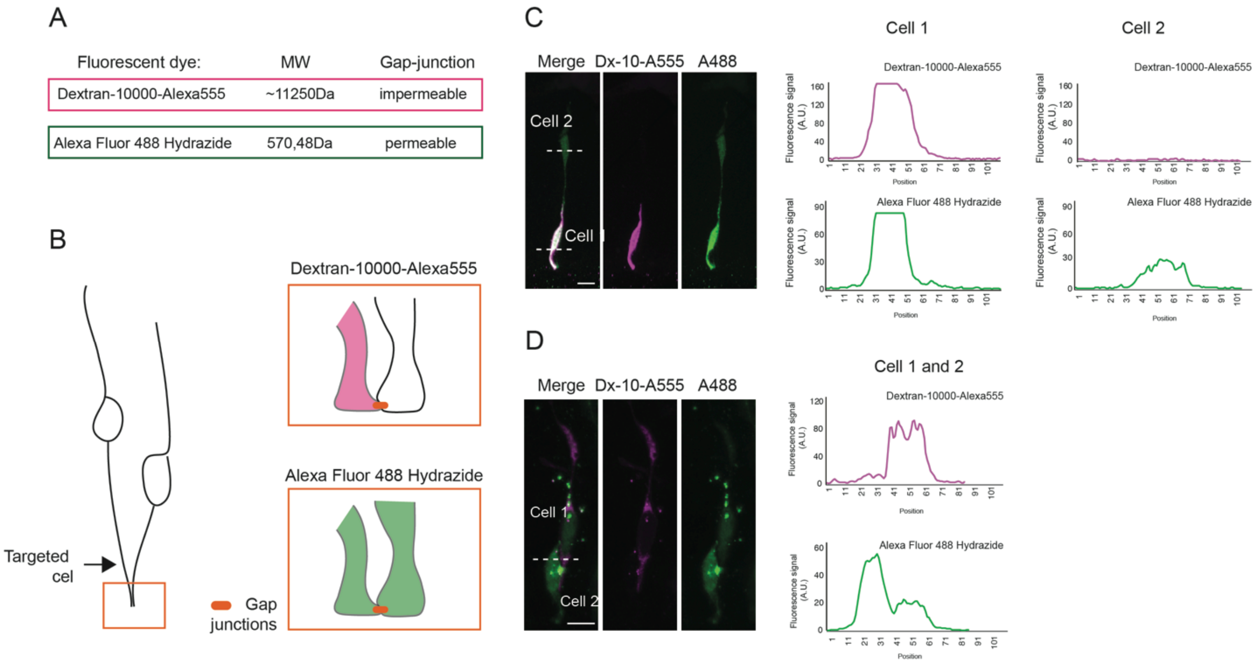

Appendix Figure 6: Use of different fluorescent dyes to visualize gap-junction-coupled clusters. Fluorescent dyes with different molecular weight used to detect gap-junctions-coupled clusters. (A) Molecular weight (MW) and gap-junction permeability of the two fluorescent dyes Dx-10-Alexa555 and Alexa-488. (B) Representative scheme of a 2-cell cluster (cartoon on left). The injected cell is positive for both the junctional impermeable dye Dx-10-Alexa555 and for the junctional permeable dye Alexa-488 (cartoon on the top right, apical endfoot, magenta), while the coupled cell is positive for the junctional permeable dye Alexa-488 only (cartoon on the bottom right, apical endfoot, green). (C, D) Images from 2-cell clusters (left) with the respective line plot quantifications of the fluorescence intensity of Dx-10-Alexa555 and Alexa-488 (right). The line plot was executed along the white dotted lines. Note that the cells shown in C are the same shown in Figure 5B. Scale bars, 10µm.

Appendix Figure S7

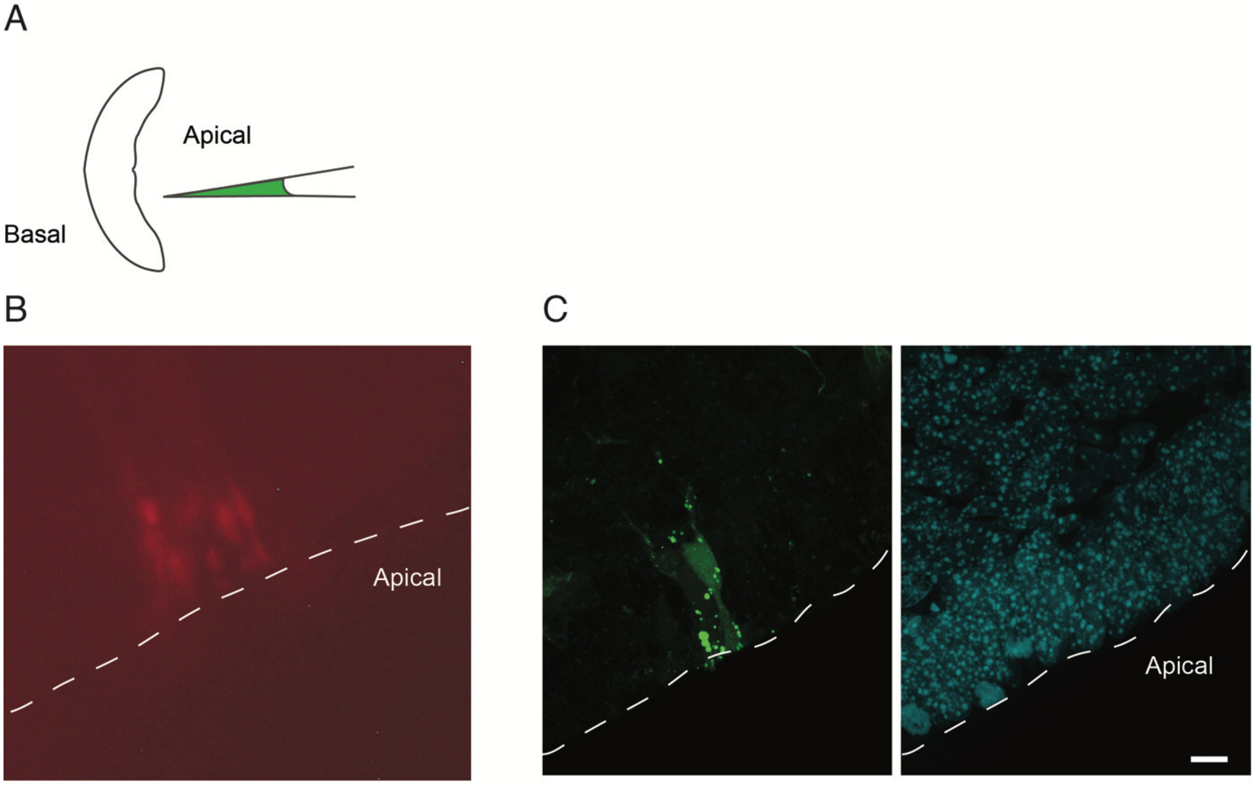

Appendix Figure 7: Automated injection of mouse hindbrain APs. Automated microinjection was performed on organotypic slices of mouse E14.5 hindbrain using Dextran-A555 (red, B) or Dextran-A488 (green, C), followed by fixation (C). (A) Schematic of hindbrain autoinjection. (B) Phase contrast picture of a hindbrain slice immediately after injection. (C) Injected slice fixed and stained for microinjection dye (green), and DAPI (cyan). In both B and C, the injected cells show the typical bipolar morphology of APs. White dotted line: apical surface. Scale bar: 10µm.

Appendix Figure S8

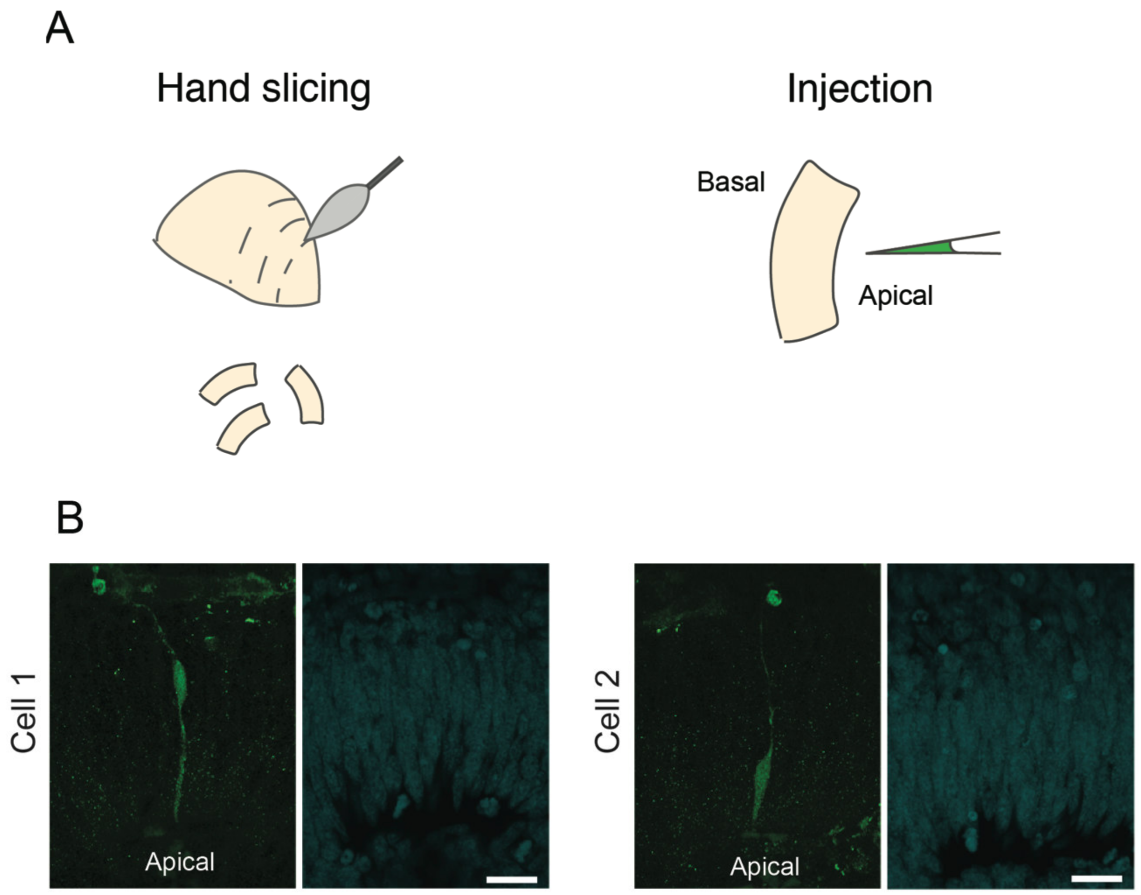

Appendix Figure 8: Automated injection of human brain APs. Automated microinjection was performed on organotypic slices of 12wpc (weeks post conception) human developing telencephalon (A, B) using Dextran-A488 (green, B), followed by fixation and DAPI staining (blue, B). (A) schematic of human tissue hand slicing (top) and auto injection (bottom). (B) two representative examples of injected APs in the human developing telencephalon. The injected cells show the typical bipolar morphology of APs. Scale bars: 20µm.

Movie Captions

Movie EV1: Image-guided automated microinjection procedure. The movie shows a microinjection into neurons via the basal lamina in the E16.5 mouse telencephalon.

Movie EV2: Injection with results. The movie shows a microinjection into single neural stem cells via the apical surface in the E14.5 mouse telencephalon. The corresponding microinjected cells are shown at the end of the movie (Dx-A555, magenta). Nuclei are stained with DAPI (cyan).

Movie EV3: Assessing cell morphology. The movie shows a confocal stack of a cell microinjected with Dx-A555 (magenta). DAPI highlights the nuclei (cyan). The image illustrates how different cellular features (arrows: apical contact, nucleus, basal process) are used to assess AP cell identity. Scale bar is 10µm.

Movie EV4: Injection Setup. This Movie shows you how to start the program, how to calibrate the autoinjector, and how to run the Autoinjector.

Movie EV5: Trouble Shooting. This Movie shows you the errors that come up when there is a hardware problem such as parts not being plugged in. The Movie suggests steps to take to fix these problems.

Movie EV6: Trouble Shooting – Draw Error. This Movie displays an error that comes up sometimes when drawing the line. If you have this error, this Movie tells you how to fix this problem.

Movie EV7: Number of Injections Executed. This Movie shows you where in the GUI to see the number of injections that were completed.

Appendix Note S1: Calibration Procedure

Movement of the micropipette using the manipulator resulted in displacement of the micropipette in the microscope camera FOV. To control the micropipette to specific locations in the microscope camera field of view (FOV), it was necessary to translate movement of the manipulator in 3D cartesian coordinate space to corresponding change in position of the micropipette in the camera FOV. This translation allowed us to guide the micropipette to any point in the FOV for targeted microinjection. To this end, we performed a calibration procedure at the beginning of each experiment.
A calibration function translated points in the camera axes (Cx, Cy) to points in the manipulator axes (Mx,My) (see Appendix Figure S2 for a representation of the calibration procedure).
We first needed to determine the angle offset between the manipulator axes and the camera axes. This was accomplished as follows:
First, the location of the micropipette tip in the camera FOV was recorded and denoted as:

$$P_1 = (C_x, C_y)$$

The manipulator was then advanced along the manipulator X-axis (M_x) by a predefined distance $|X|$, resulting in a final position of the micropipette tip in the camera FOV, recorded and denoted as:

$$P_2 = (C_x, C_y)$$

The distance in pixels between P_1 and P_2 was given by:

$$D(C_x, C_y) = \left|P_2(C_x, C_y) \text{-} P_1(C_x, C_y)\right|$$

The angle between the manipulator X-axis (Mx) and the camera X-axis Cx was given by the equation:

$$\theta = \tan^{-1} \frac{D(C_x)}{D(C_y)}$$

The absolute distance (D) between P1 and P2 was then calculated using the equation:

$$\mathrm{D} = \sqrt{D(C_x)^2 + D(C_y)^2}$$

The scaling factor (S) between manipulator distance and camera pixels was calculated as S:

$$\mathrm{S} = \frac{\mathrm{D}}{|X|}$$

The equation to relate the camera axes to manipulator axes was given by:

$$\begin{bmatrix} C_x \\ C_y \end{bmatrix} = R(\theta) \begin{bmatrix} M_x \\ M_y \end{bmatrix}$$

Where:

$$R(\theta) = \begin{bmatrix} \text{-}S\cos\theta & S\sin\theta \\ \text{-}S\sin\theta & \text{-}S\cos\theta \end{bmatrix}$$

Solution for the manipulator axes in terms of the camera axes was obtained using the equations:

$$\begin{bmatrix} M_x \\ M_y \end{bmatrix} = R^{-1}(\theta) \begin{bmatrix} C_x \\ C_y \end{bmatrix}$$

Where

$$R^{-1}(\theta) = \frac{1}{S^2} \begin{bmatrix} -S\cos\theta & -S\sin\theta \\ S\sin\theta & -S\cos\theta \end{bmatrix}$$

Thus, M_x and M_y were given by:

$$M_x = \frac{1}{S}\left(-C_x \cos\theta - C_y \sin\theta\right)$$

And

$$M_y = \frac{1}{S}\left(C_x \sin\theta - C_y \cos\theta\right)$$

Using these equations, any point in the camera axes (C_x, C_y) could be converted to a point in the manipulator axes (M_x,M_y) for image guided position control of the injection micropipette.

Appendix Note S2: Trajectory Generation

The Autoinjector software allowed the user to define a path of microinjection, and to customize the trajectory of microinjection using user defined parameters (see **Appendix Fig. 2B**). These include: the depth the micropipette is inserted into tissue during microinjection, denoted as D, the distance the micropipette pulls out of the tissue after microinjection attempts, denoted as A, the spacing along the path between subsequent microinjection attempts, denoted as S, the speed the micropipette is inserted into the tissue during the whole procedure, defined as percentage of the maximum speed the manipulator was capable of performing at (1 mm/s), These parameters were utilized to generate the path for the micropipette to take during microinjection was created according to the following protocol:

The user traced a path using the cursor on the camera image along the desired path of microinjection, during which a set of points at different pixels along this path were recorded, This set of pointed was denoted as $t(C_x, C_y)$. A univariate spline interpolation [1,2] was used to connect these points into a continuous path. The interpolated path was denoted as $T(C_x, C_y)$.

Using the calibration procedure (see **Appendix Note S1**), $T(C_x, C_y)$ was transformed to a trajectory in the manipulator coordinate space. This was denoted as $T(M_x, M_y)$:

$$\begin{bmatrix} T(M_x) \\ T(M_y) \end{bmatrix} = R^{-1}(\theta) \begin{bmatrix} T(C_x) \\ T(C_y) \end{bmatrix}$$

Thus, $T(M_x)$ and $T(M_{y)}$ were given by:

$$T(M_x) = \frac{1}{S}\left(-T(C_x)\cos\theta - T(C_y)\sin\theta\right)$$

and

$$T(M_y) = \frac{1}{S}\left(T(C_x)\sin\theta - T(C_y)\cos\theta\right)$$

Where $R(\theta)$ was the calibration matrix described in **Appendix Note S1.**

To compute the final trajectory of microinjection, the coordinates for all injection sites was denoted as:

$$T_i(M_x, M_y) = \begin{bmatrix} T_1(M_x, M_y) \\ T_2(M_x, M_y) \\ \cdots \\ \cdots \\ T_N(M_x, M_y) \end{bmatrix}$$

where and N is the total number of injection attempts. For each point i, the injection micropipette first went to the position $T_i(M_x, M_y)$. The injection micropipette was then moved to $T_i(M_x + D, M_y)$ where D was the user specified the depth of injection. The injection pipette was then moved to $T_i(M_x + D\text{-}A, M_y)$, resulting in a retraction of the micropipette by a distance A. The micropipette was then moved to $T_i(M_x + D, M_y\text{-}s)$, where s was the user defined inter injection site spacing. This process was repeated until the end of the drawn line was reached.

Appendix Note S3: Calibration consistency across users

Visual detection of pipette tip during the calibration can depend on individual perception and can subsequently be a source of positioning error of the Autoinjector. We examined user variability by having four independent users perform the calibration which solves for the angle offset between the manipulator axes and the camera axes. One of the users was an expert user of the Autoinjector, and the other three users had no prior experience calibrating the device. The angle offset calculated from the expert user's calibration was found to be 2.99 ± 0.163 degrees (n = 3 calibrations). The angle offset values calculated from the inexperienced user's calibrations were found to be 2.64 ± 0.223 degrees for inexperienced user 1, 2.86 ± 0.382 degrees for inexperienced user 2, and 2.73 ± 0.341 degrees for inexperienced user 3. Significance tests were performed using a Mann-Whitney U-test indicated that there was no significant difference between the experienced user and inexperienced user 1 (p = 0.400), inexperienced user 2 (p = 0.700), nor inexperienced user 3 (p = 0.800). To determine the effects of user calibration on resolution, we performed a resolution test described in the main text (except for here using 4 points rather than 8) using the inexperienced user's calibration angle with the highest standard deviation (inexperienced user 2, calibration angle = 2.86 ± 0.382 degrees). The inexperienced user 2 had an average error in the manipulator axes for the x, and y axis of 0.524 ± 0.409 µm, and 0.518 ± 0.480 µm, respectively (n = 5 measurements per point, 4 points). This value was deemed sufficient for experiments as the distances used in experiments are much greater than this resolution value. Thus, the accuracy obtained across experimenters indicated that variation in user's visual perception does not significantly affect calibration angles.

Appendix Table S1: Parts and Reagents

REAGENT or RESOURCE	SOURCE	IDENTIFIER
**Antibodies**		
Chicken anti-Tbr2	Merck Millipore	Cat# AB15904, RRID: AB_10615604
Goat anti SOX2	R&D Systems	Cat# AF2018, RRID: AB_355110
Rabbit anti-Tbr2	Abcam	Cat# ab222226, RRID: AB_2721040
Rabbit anti-Tbr2	Abcam	Cat# ab183991, RRID: AB_2721040
Rabbit anti-RFP	Rockland antibodies	Cat# 600-401-379, RRID: AB_2209751
Rabbit anti-g-tubulin	Sigma-Aldrich	Cat# T5192, RRID: AB_261690
Mouse anti-g-tubulin	Sigma-Aldrich	Cat# T6557, RRID: AB_477584
Mouse anti Satb2	Abcam	Cat# ab51502, RRID: AB_882455
Mouse anti-TUJ1	BioLegend	Cat# 801201, RRID: AB_2313773
Mouse anti-TUJ1, Alexa Fluor®488	BioLegend	Cat# 801203, RRID: AB_2564757
Mouse anti-TUJ1, Alexa Fluor®647	BioLegend	Cat# 801209, RRID: AB_2686930
Mouse anti-Dextran	STEMCELL Technologies	Cat# 60026, RRID: AB_2651016
Mouse Anti-Dextran, FITC	STEMCELL Technologies	Cat# 60026FI, RRID: AB_2651016
Mouse anti Pax6	Biolegend	Cat# 901301, RRID: AB_2565003
Mouse anti-Reelin	Abcam	Cat# ab78541, RRID: AB_1603148
Lucifer Yellow Polyclonal Antibody	Thermo Fisher Scientific	Cat# A-5750, RRID: AB_2536190
Alexa Fluor 488 Polyclonal Antibody	Thermo Fisher	Cat# A-11094, RRID: AB_221544
Goat anti-Chicken IgY (H+L) Secondary Antibody, Alexa Fluor 647	Thermo Fisher Scientific	Cat# A-21449, RRID: AB_2535866
Donkey anti-Mouse IgG (H+L) Secondary Antibody, Alexa Fluor A555	Thermo Fisher Scientific	Cat# A-31570, RRID: AB_2536180
Donkey anti Mouse IgG (H+L) Secondary Antibody, Alexa Fluor A488	Thermo Fisher Scientific	Cat# A-21202, RRID: AB_141607
Donkey anti Mouse IgG (H+L) Secondary Antibody Alexa Fluor A647	Thermo Fisher Scientific	Cat# A-31571, RRID: AB_162542

Donkey anti Rabbit IgG (H+L) Secondary Antibody, Alexa Fluor A488	Thermo Fisher Scientific	Cat# A-21206, RRID: AB_2535792
Donkey anti Rabbit IgG (H+L) Secondary Antibody, Alexa Fluor A647	Thermo Fisher Scientific	Cat# A-31573, RRID: AB_2536183
Donkey anti Rabbit IgG (H+L) Secondary Antibody Alexa Fluor A555	Thermo Fisher Scientific	Cat# A-31572, RRID: AB_162543
Donkey anti Goat IgG (H+L), Secondary Antibody Alexa Fluor A488	Thermo Fisher Scientific	Cat# A-11055, RRID: AB_2534102
Donkey anti Goat IgG (H+L), Secondary Antibody Alexa Fluor A555	Thermo Fisher Scientific	Cat# A-21432, RRID: AB_2535853
Donkey anti Goat IgG (H+L), Secondary Antibody Alexa Fluor A647	Thermo Fisher Scientific	Cat# A-21447, RRID: AB_141844
**Bacterial and Virus Strains**		
One Shot TOP10 Chemically Competent E.coli	Thermo Fisher Scientific	Cat# C404010
**Biological Samples**		
12 wpc human fetal neocortical tissue	Tissue obtained with informed written maternal consent, followed by elective pregnancy termination from Klinik und Poliklinik für Frauenheilkunde und Geburtshilfe, Universitätsklinikum Carl Gustav Carus of the Technische Universität Dresden	N/A
**Chemicals and Peptides**		
DAPI (4′,6-Diamidine-2′-phenylindole dihydrochloride)	Thermo Fisher Scientific	Cat# 000000010236276001
Alexa Fluor 647 Phalloidin	Thermo Fisher Scientific	Cat# A22287
Alexa Fluor 488 Phalloidin	Thermo Fisher Scientific	Cat# A12379
Alexa Fluor 488 Hydrazide	Thermo Fisher Scientific	Cat# A10436
Cdc42-GST Protein Wild-type	Cytoskeleton, Inc.	Cat# CDG01
Dextran, Alexa Fluor 488; 3,000 MW, Anionic	Thermo Fisher Scientific	Cat# D34682
Dextran, Alexa Fluor 488; 10,000 MW, Anionic	Thermo Fisher Scientific	Cat# D22910
Dextran, Alexa Fluor 555; 10,000 MW, Anionic	Thermo Fisher Scientific	Cat# D34679
Lucifer Yellow CH, Lithium Salt, for microinjection	Cat# L12926	
mMessage mMachine T7 Ultra Transcription Kit	Cat# AM1345	
Agarose, Low Melt	Carl Roth	Cat# 6351.2

Agarose, Wild Range	Sigma-Aldrich	Cat# A2790
B-27 Supplement	Thermo Fisher Scientific	Cat# 17504044
Cellmatrix Type-IA (Collagen, Type !)	FUJIFILM Wako Chemicals	Cat# 637-00653
Distilled Water		
DMEM-F12, CO2 independent (w/o Phenol red)	Sigma-Aldrich	Cat# D2906
DMEM-F12, CO2 independent (with Phenol red)	Sigma-Aldrich	Cat# D8900
HEPES-NAOH, pH 7.2, 1M (HEPES buffer)	Carl Roth	Cat# 9105.3
L-Glutamine, 200 mM	Thermo Fisher Sientific	Cat# 25030024
Click-iT^{TM} EdU Alexa Fluor^{TM} 647 Imaging Kit	Thermo Fisher Scientific	Cat# C10340
Liquid Nitrogen	AirLiquide	
Mowiol 4-88	Sigma-Aldrich	Cat# 81381
N-2 Supplement	Thermo Fisher Scientific	Cat# 17502048
Neurobasal Medium	Thermo Fisher Scientific	Cat# 21103049
Nuclease-free water	Thermo Fisher Scientific	Cat# AM9937
Rat serum	Charles River Laboratories Japan	
Best-CA 221 Glue	Best Klebstoffe GmbH & Co.KG	Cat# CA221-10ml
Sodium bicarbonate (NaHCO3)	Merck	Cat# 106323
Sodium hydroxide (NaOH)	Merck	Cat# 106482
Tyrode's salt	Sigma	Cat# T2145-10x1L)
Paraformaldehyde	Merck	Cat# 818715
Penicillin-Streptomycin (10,000 U/ml)	Thermo Fisher Scientific	Cat# 15140122
PBS		
O2 (40%), CO2 (5%), N2 (55%) Mix, 50 liters		
**Experimental Models: Organism/Strains**		
Mouse: C57BL/6JOlaHsd	Envigo	N/A
Recombinant DNA	Taverna et al., 2001	
pTNT-mRFP		N/A
**Equipment**		
Borosilicate glass capillaries, 1.2 mm outer diameter x 0.94 mm inner diameter	Sutter Instruments	Cat# BF-120-94-10
Bottle-top filter system, 500 ml	Corning	Cat# 430769
Light source	Olympus	Cat# Highlight 3100
Falcon tubes, 15 ml	Corning	Cat# 430791
Falcon tubes, 50 ml	Corning	Cat# 430829
Fine-tip paintbrush		
Flaming/ Brown micropipette puller	Sutter Instruments	Cat# P-97

Forceps, Dumont no. 3	Fine Science Tools	Cat# 11231-30
Forceps, Dumont no. 5	Fine Science Tools	Cat# 11255-20
Forceps, Dumont no. 55	Fine Science Tools	Cat# 11252-20
Microloader Tips	Eppendorf	Cat# 5242956.003
Heating block	Labtech International	Cat # Dri block Digi2
Inverted fluorescence microscope	Zeiss	Cat# Axiovert 200
Motorized stage XY for microscope		
Three-axis Manipulator	Sensapex Inc	Cat# tree-axis uMP
Microcontroller	Arduino	Cat# Arduino Due
Microscope camera Hamamatsu Orca Flash 4.0 V3		
**Custom pressure rig**		
Manual pressure regulator	McMaster Carr	Cat# 0-60 PSI 41795K3
Electronic pressure regulator	Parker Hannifin	Cat# 990-005101-002
Solenoid valve		Cat# LHDA053321H-A
Computer PC		
Pipette holder		Cat# 64-2354 MP-s12u
Multiwell plate, 24 wells	Nunc	Cat# 142475
Pasteur pipettes, plastic		
Pipette and tips		
Petri dish, 60 x 15 mm	Greiner	Cat# 628102
Petri dish, 35 x 10 mm	Nunc	Cat# 153066
Petri dish, 34 x 14 mm, including Microwell no. 1.5 cover glass	Cat# P35G-1.5-14-C	
Puller filament, 3.0-mm square box filament		Cat# FB330B
Slice culture incubation box	MPI-CBG	Cat# custom made
Stereomicroscope		Cat# SZX12
Tabletop centrifuge		Cat# 5431622
Thermometer		
Vibratome		Cat# VT1000s
Whole-embryo-culture-system incubator		Cat# RKI-10-0310
Waterbath		
**Software and Algorithms**		
Fiji		RRID: SCR_002285
Python	Python Software foundation	Python 2.7.12
Arduino	Arduino	
ZEN		RRID: SCR_013672

References

1. Jones E, Oliphant E, Peterson P, et al. SciPy: Open Source Scientific Tools for Python, 2001-, http://www.scipy.org/
2. "scipy.interpolate.UnivariateSpline." SciPy: Open Source Scientific Tools for Python, https://docs.scipy.org/doc/scipy/reference/generated/scipy.interpolate.UnivariateSpline.html
