# Supplementary material for: Robotic platform for microinjection into single cells in brain tissue
Source: EMBO Rep. 2019 Aug 30;20(10):e47880. doi: 10.15252/embr.201947880 (PMC6776899; doi:10.15252/embr.201947880)
Supplement: Supplementary file 2 — Expanded View Figures PDF [file EMBR-20-e47880-s002.pdf]

Expanded View Figures

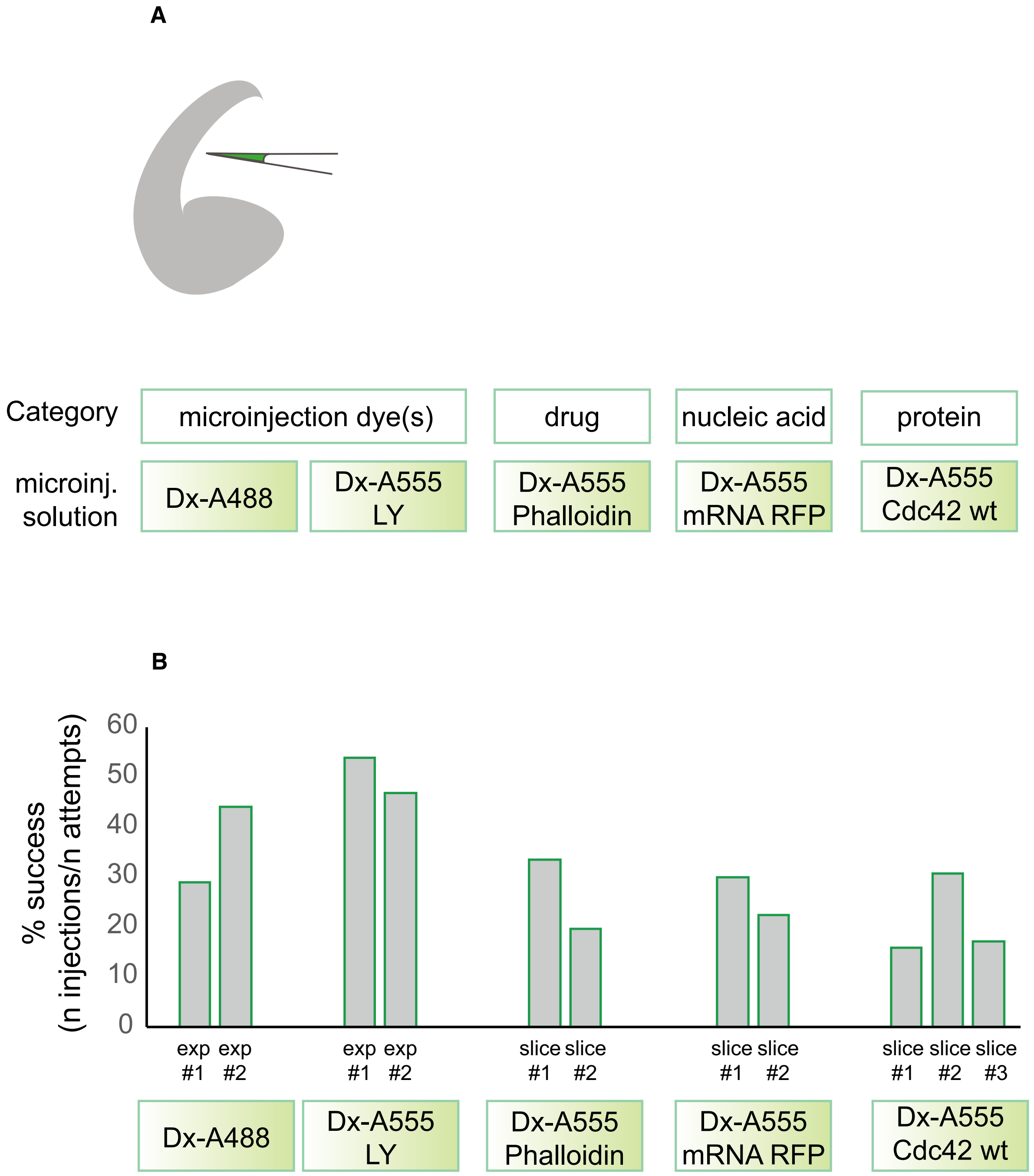

Figure EV1. Autoinjector performance using different chemicals.

A Top: schematic of telencephalon microinjected with different classes of chemicals. Bottom: list of chemicals that were tested (microinjection solution), divided by category.

B Successful injections represented as a percentage of total all chemicals. The results are divided by experiment and by slice to show the intrinsic variability of the system.

Source data are available online for this figure.

© 2019 The Authors

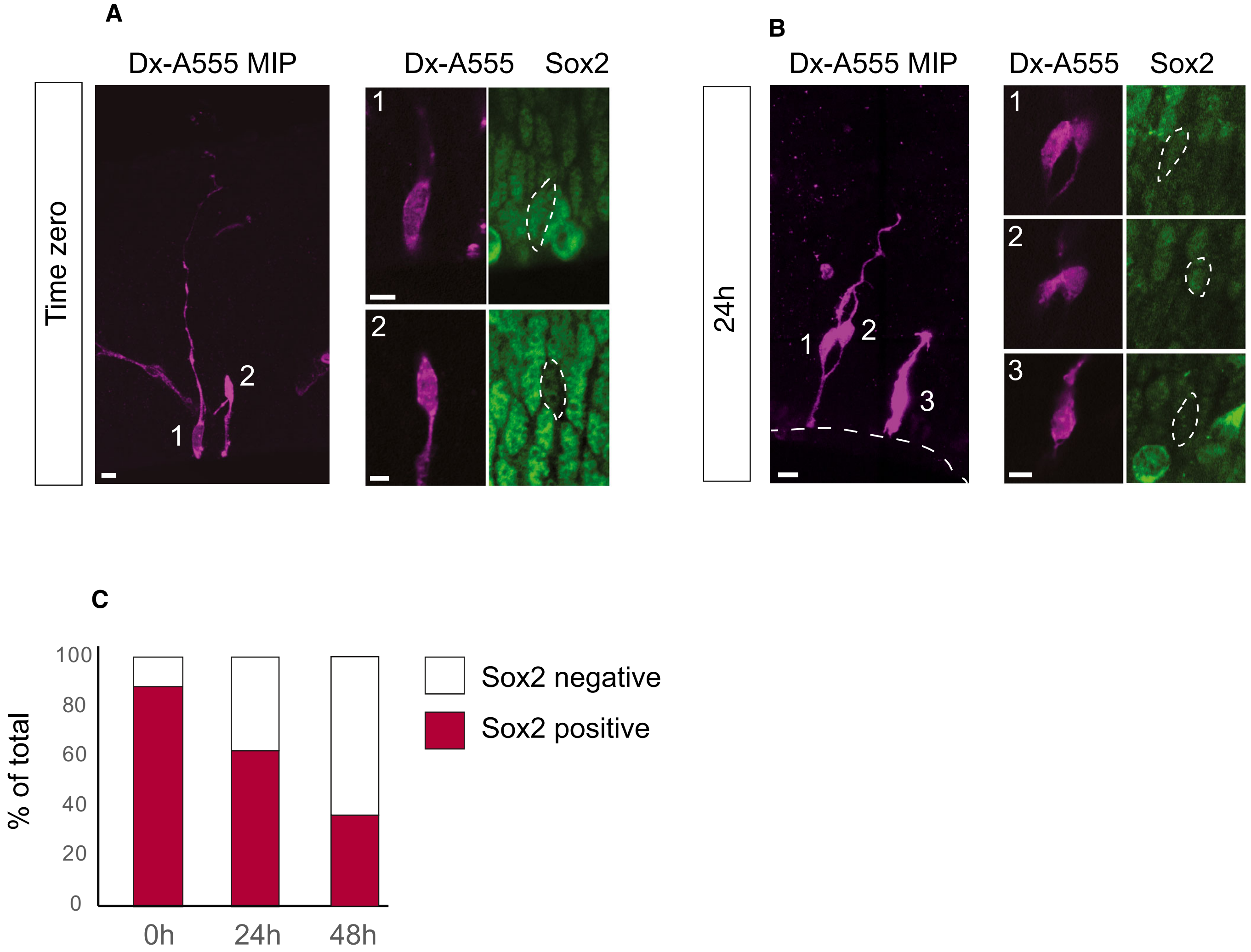

Figure EV2. Neural stem cell lineage tracing: Sox2 expression.

Automated microinjection was performed on organotypic slices of mouse E14.5 dorsal telencephalon using Dextran-A555 (A, B) without (0 h, A) or with slice culture for 24 or 48 h (24 h, B, C). After fixation, slices were stained for Sox2.

- A Microinjected cells at 0 h; injected cells show the typical bipolar morphology of an AP and are mainly positive for the AP marker Sox2. Note that the cell on the left shows a prominent basal process that reaches the basal lamina, while the cell on the right does not feature any basal process, and it is most likely a short neural precursor (SNP).
- B Cell progeny 24 h after microinjection; cell 1 is mild positive, cell 2 is positive, and cell 3 is negative for Sox2.
- C Expression of Sox2 in microinjected cells and their progeny at 0, 24, and 48 h (0 h n = 83 cells in total from three independent experiments, 24 h n = 53 cells in total from three independent experiments, 48 h n = 21 cells in total from two independent experiments).

Data information: In (A, B), the images on the left are maximum intensity projections (MIP) of 30 and 25 focal planes, respectively; images on the right are single optical sections corresponding to the nucleus area; scale bars are 20 μm for MIP and 10 μm for single focal planes.

Source data are available online for this figure.

 © 2019 The Authors
