# Supplementary material for: Robotic platform for microinjection into single cells in brain tissue
Source: EMBO Rep. 2019 Aug 30;20(10):e47880. doi: 10.15252/embr.201947880 (PMC6776899; doi:10.15252/embr.201947880)
Supplement: Supplementary file 3 — Movie EV1 [file EMBR-20-e47880-s003.zip › 47880V2_Movie_EV1_caption.docx]

**Movie EV1: Image-guided automated microinjection procedure**. The movie shows a microinjection into neurons via the basal lamina in the E16.5 mouse telencephalon.
