# Supplementary material for: Robotic platform for microinjection into single cells in brain tissue
Source: EMBO Rep. 2019 Aug 30;20(10):e47880. doi: 10.15252/embr.201947880 (PMC6776899; doi:10.15252/embr.201947880)
Supplement: Supplementary file 4 — Movie EV2 [file EMBR-20-e47880-s004.zip › 47880V2_Movie_EV2_caption.docx]

**Movie EV2: Injection with results.** The movie shows a microinjection into single neural stem cells via the apical surface in the E14.5 mouse telencephalon. The corresponding microinjected cells are shown at the end of the movie (Dx-A555, magenta). Nuclei are stained with DAPI (cyan).
