# Supplementary material for: Robotic platform for microinjection into single cells in brain tissue
Source: EMBO Rep. 2019 Aug 30;20(10):e47880. doi: 10.15252/embr.201947880 (PMC6776899; doi:10.15252/embr.201947880)
Supplement: Supplementary file 5 — Movie EV3 [file EMBR-20-e47880-s005.zip › 47880V2_Movie_EV3_caption.docx]

**Movie EV3: Assessing cell morphology.** The movie shows a confocal stack of a cell microinjected with Dx-A555 (magenta). DAPI highlights the nuclei (cyan). The image illustrates how different cellular features (arrows: apical contact, nucleus, basal process) are used to assess AP cell identity. Scale bar is 10µm.
