# Supplementary material for: Robotic platform for microinjection into single cells in brain tissue
Source: EMBO Rep. 2019 Aug 30;20(10):e47880. doi: 10.15252/embr.201947880 (PMC6776899; doi:10.15252/embr.201947880)
Supplement: Supplementary file 6 — Movie EV4 [file EMBR-20-e47880-s006.zip › 47880V2_Movie_EV4_caption.docx]

**Movie EV4: Injection Setup.** This Movie shows you how to start the program, how to calibrate the autoinjector, and how to run the Autoinjector.
