# Supplementary material for: Robotic platform for microinjection into single cells in brain tissue
Source: EMBO Rep. 2019 Aug 30;20(10):e47880. doi: 10.15252/embr.201947880 (PMC6776899; doi:10.15252/embr.201947880)
Supplement: Supplementary file 7 — Movie EV5 [file EMBR-20-e47880-s007.zip › 47880V2_Movie_EV5_caption.docx]

**Movie EV5: Trouble Shooting.** This Movie shows you the errors that come up when there is a hardware problem such as parts not being plugged in. The Movie suggests steps to take to fix these problems.
