# Supplementary material for: Robotic platform for microinjection into single cells in brain tissue
Source: EMBO Rep. 2019 Aug 30;20(10):e47880. doi: 10.15252/embr.201947880 (PMC6776899; doi:10.15252/embr.201947880)
Supplement: Supplementary file 8 — Movie EV6 [file EMBR-20-e47880-s008.zip › 47880V2_Movie_EV6_caption.docx]

**Movie EV6: Trouble Shooting – Draw Error.** This Movie displays an error that comes up sometimes when drawing the line. If you have this error, this Movie tells you how to fix this problem.
