# Supplementary material for: Robotic platform for microinjection into single cells in brain tissue
Source: EMBO Rep. 2019 Aug 30;20(10):e47880. doi: 10.15252/embr.201947880 (PMC6776899; doi:10.15252/embr.201947880)
Supplement: Supplementary file 9 — Movie EV7 [file EMBR-20-e47880-s009.zip › 47880V2_Movie_EV7_caption.docx]

**Movie EV7: Number of Injections Executed.** This Movie shows you where in the GUI to see the number of injections that were completed.
