# Supplementary material for: Robotic platform for microinjection into single cells in brain tissue
Source: EMBO Rep. 2019 Aug 30;20(10):e47880. doi: 10.15252/embr.201947880 (PMC6776899; doi:10.15252/embr.201947880)
Supplement: Supplementary file 11 — User Manual and Guidelines [file EMBR-20-e47880-s015.pdf]

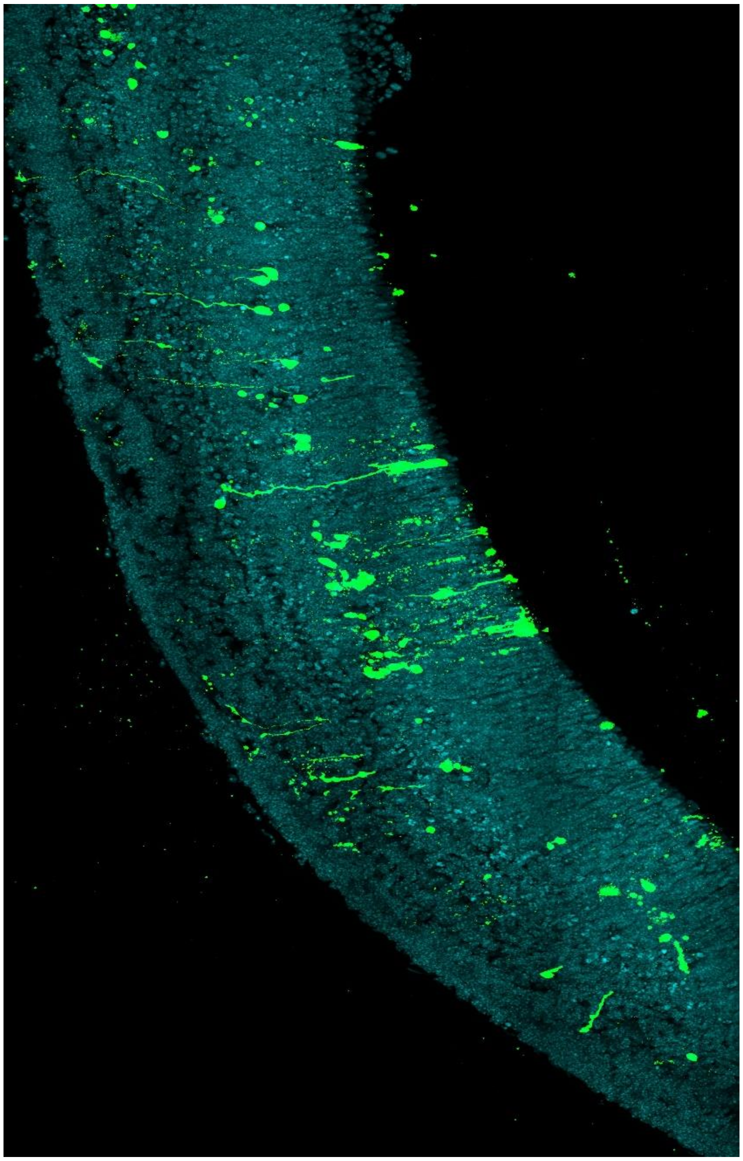

Automated Microinjection

USER MANUAL AND GENERAL GUIDELINES

Last Updated: June 2019

Contents

I. Introduction 2
II. Hardware Overview 2
Photo Schematic of hardware 3
sCircuit Diagram 3
III. Software Overview 4
IV. Turning on Devices 6
V. Running Experiment 8
VI. Trouble Shooting 16
VI. Movie Tutorial Descriptions 17
VII. Guidelines 18
Preparing for microinjection 20
How to obtain a good pipette 20
The chemical nature of the microinjection solution and its effects on the efficiency of injection 20
Microinjection 21
Determining inter-injection spacing 21
Efficiency of translation for mRNA microinjection 22

If you have any questions you can reach out to:

Suhasa B Kodandaramaiah - suhasabk@umn.edu

Elena Taverna - taverna@mpi-cbg.de

Christiane Haffner - haffner@mpi-cbg.de

Gabriella Shull - gms46@duke.edu

I. Introduction

General microinjection guidelines are found in section VII and provide suggestions for parameter values. This user manual is designed to take you through the implementation of the automated microinjection system with the following assumptions:

- Software is installed from github.com/ogshull/Autoinjector-
- You are using Sensapex manipulators, the custom pressure rig, and a Hamamatsu camera

II. Hardware Overview

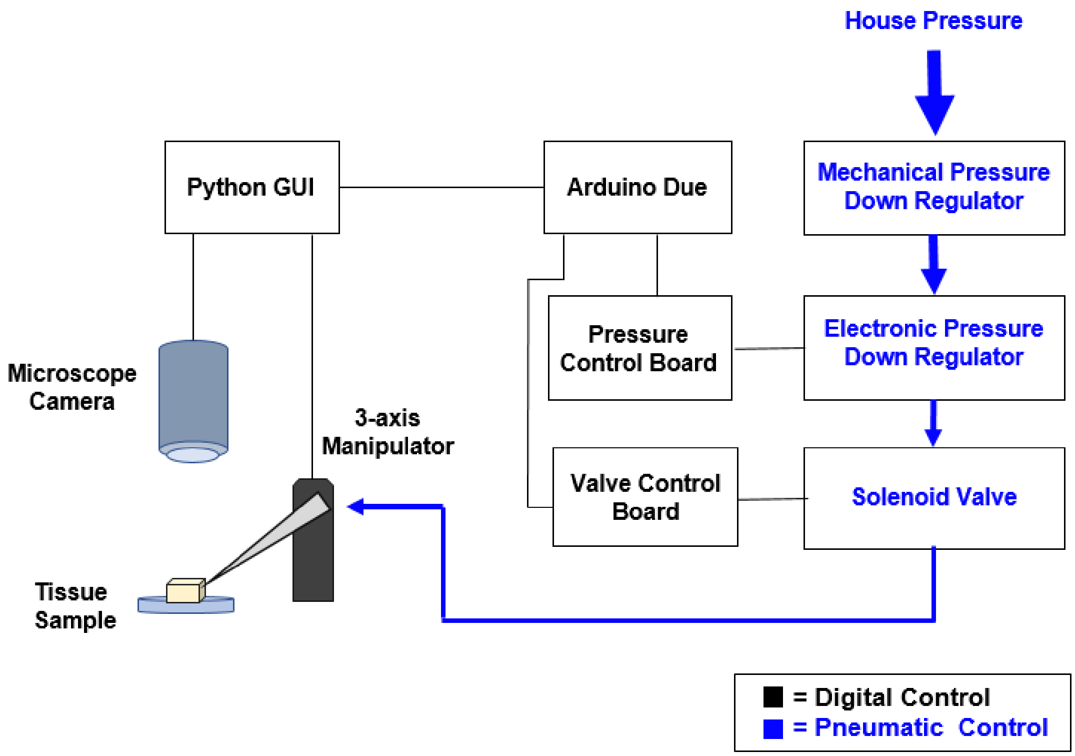

The image above provides an overview of how the Autoinjector system operates with digital control shown in black and pneumatic (pressure) control shown in blue. All hardware components are controlled by the python guide user interface from the computer (GUI). In summary, the pressure is set by the user in the software which sends a signal to the Arduino (microcontroller) which in turn sets the pressure via the pressure control board and opens the valve (further discussion in section 5). The following page displays an in-depth diagram and a schematic of the hardware.

PHOTO SCHEMATIC OF HARDWARE

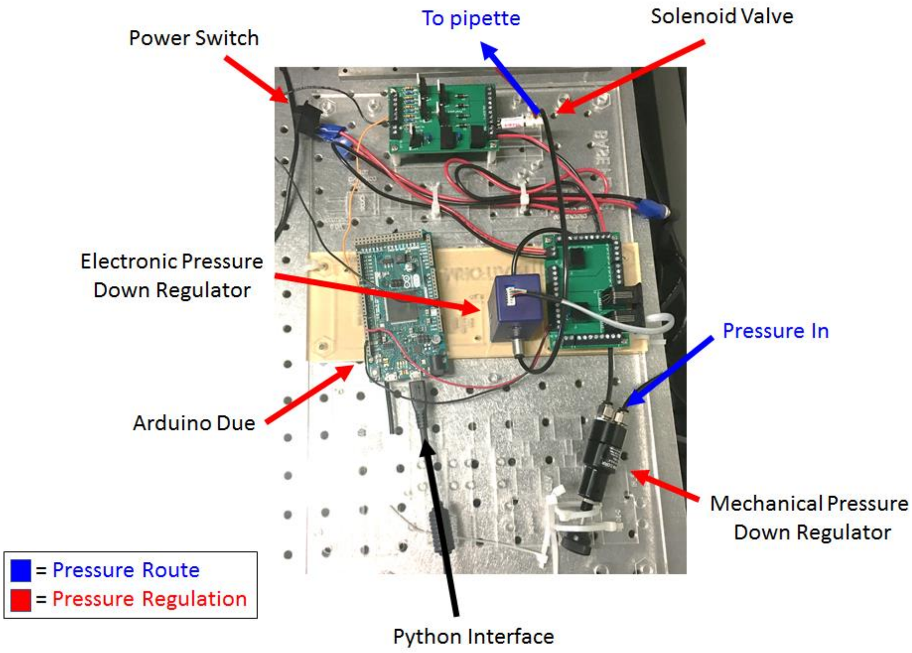

CIRCUIT DIAGRAM

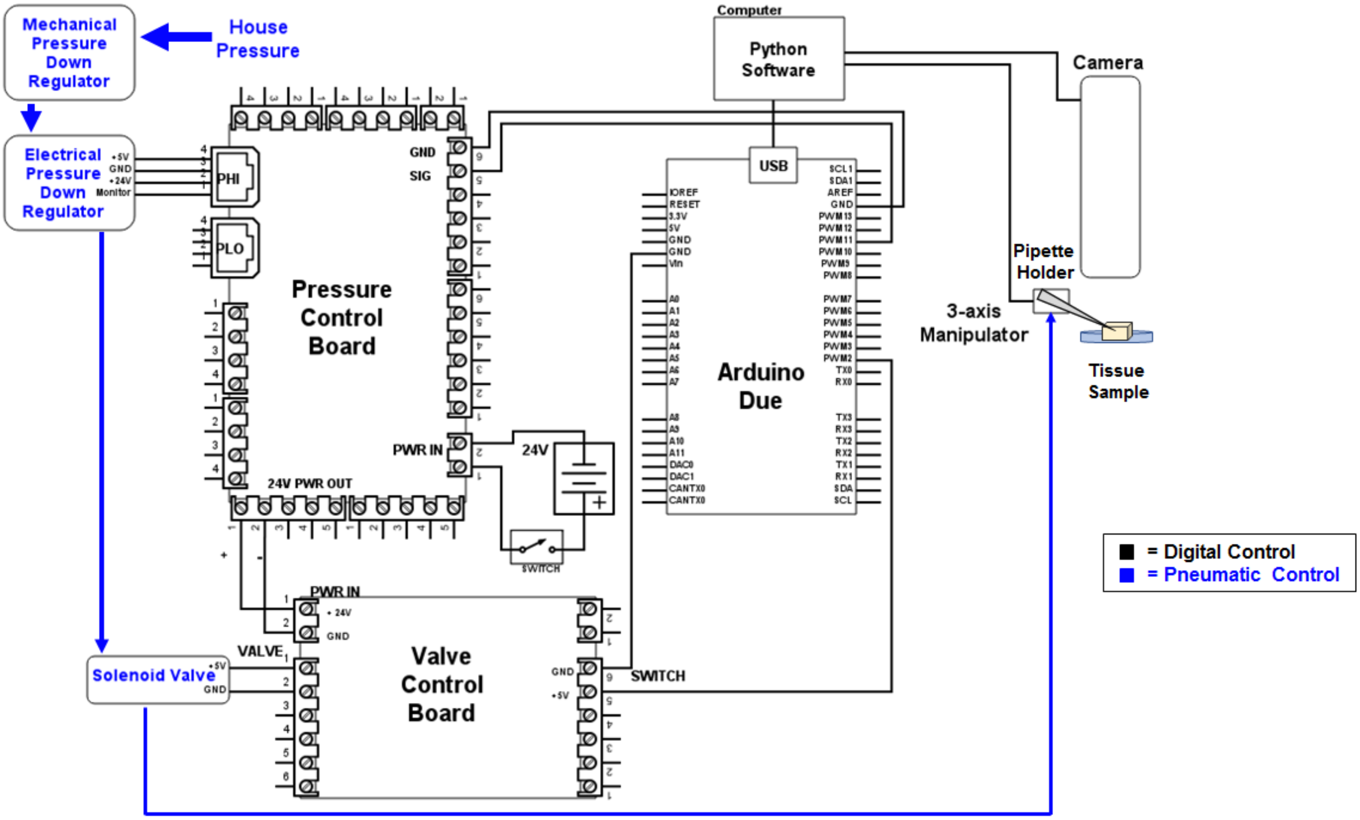

III. Software Overview

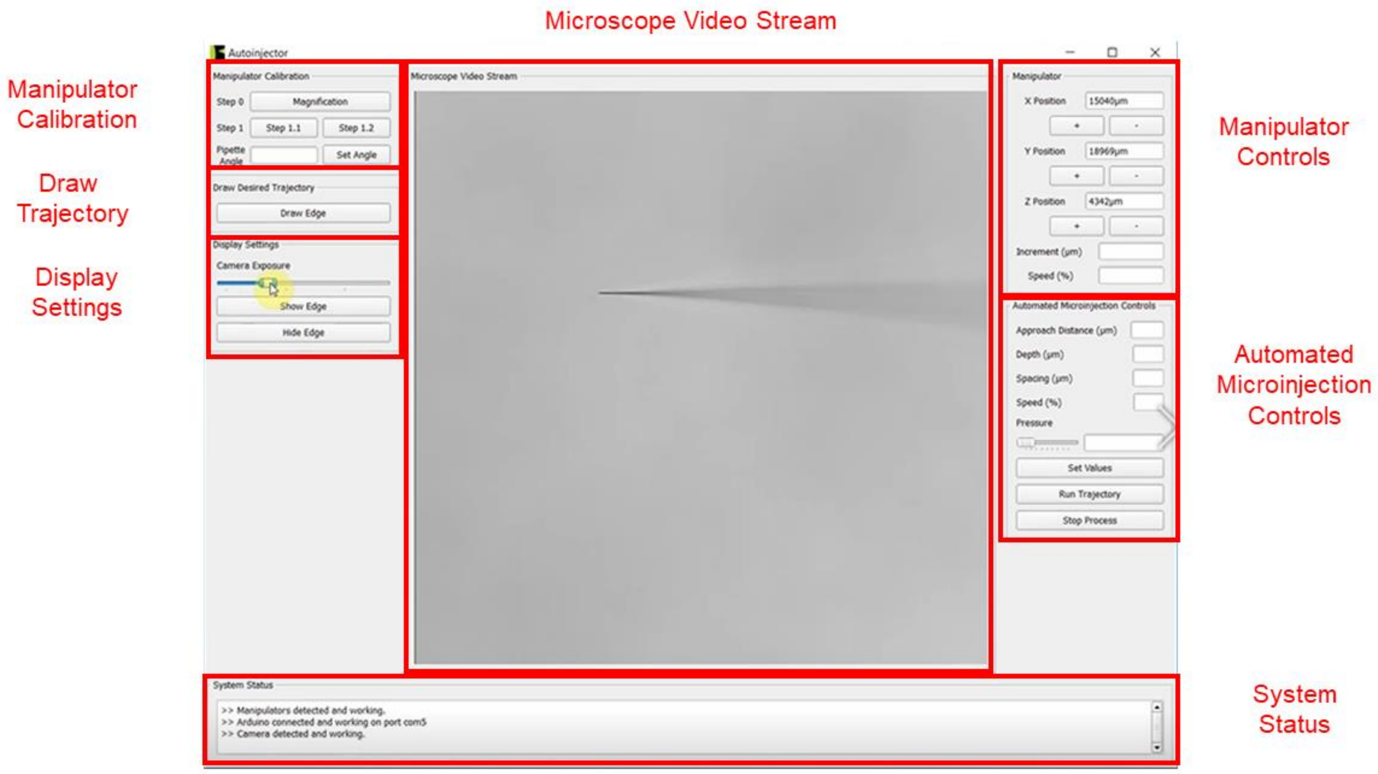

The image above displays the guide user interface (GUI) written in python and can be launched from the desktop of the computer (see section V step 3 for further details). The parts are as follows:

1. **Manipulator Calibration** – the calibration controls allow the user to calibrate the manipulator camera frames to those of the camera. Further instructions can be found in section V).

2. **Draw Trajectory** – This is composed of the "Draw Edge" Button which allows the user to draw the desired trajectory (see section V steps 15 – 18).

3. **Display Settings** - This allows you to control the gain of the microscope feed, and display or hide the trajectory you draw from the draw trajectory section

4. **System Status** - The system status updates the user with feedback from the interface and will report useful information such as parameter change updates, and errors.

5. **Manipulator Controls -** The manipulator controls display the positions of the manipulator and allow you to advance the axes from the interface. Set the increment (in microns) and speed (in % total, 100% is fine in most cases) by typing the values into the interface and press the "+" or "-" buttons. If the numbers do not appear upon starting the interface see trouble shooting section for further assistance, this indicates a manipulator error and will require you to restart the program.

6. **Automated Microinjection Controls** - The trajectory controls are as follows (see section V for parameter selection values):

   - ❖ *Approach Distance* – the distance the pipette pulls out of the tissue before advancing to next injection site in microns.
   - ❖ *Depth* - the axial depth into the tissue the pipette goes upon injection in microns.
   - ❖ *Spacing* – the spacing between subsequent injections in microns.
   - ❖ *Speed* – the speed of the manipulator in microns/s

7. **Microscope Video Stream –** The images from the microscope camera are displayed here as a live video stream.

IV. Turning on Devices

Before the experiment turn on the following devices (it does not matter what order you turn on the devices). It is recommended you turn on the devices at least 10 minutes before you intend to do a microinjection.

1. **Turn on microscope**

2. **Turn on the Computer**

3. **Turn on the Sensapex Manipulators**
    a. Hold down the button on the Sensapex screen until it lights up as pointed to in the arrow in the image below.
    b. The Sensapex manipulators are connected to the computer via ethernet. Make sure the black ethernet cable is connected to the back of the PC as shown in the image below to the right.

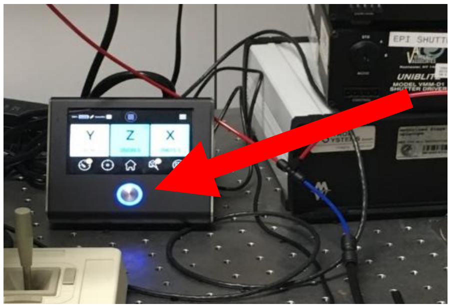

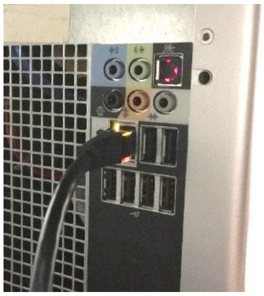

4. **Turn on the Pressure Rig Switch**
    a. The pressure rig is shown on the images below. Turn on the power rig by flipping the switch (zoomed in image on right). The image below zoomed in shows the power supply switch in the 'ON' state.

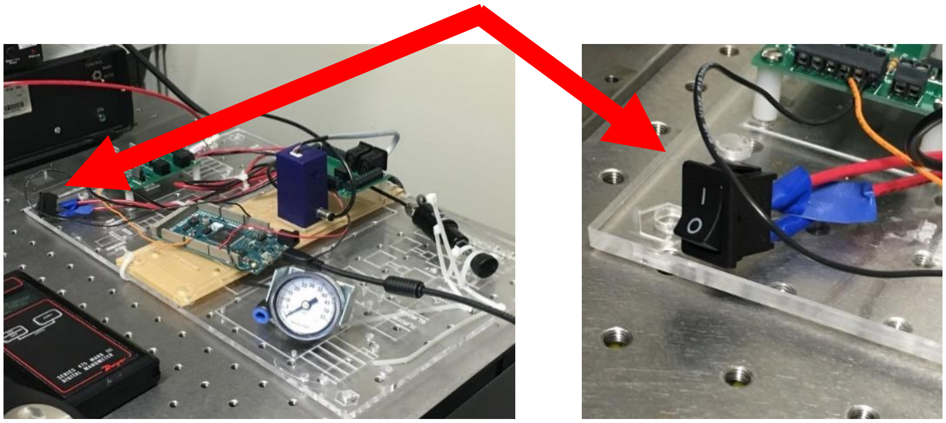

5. **Turn on the camera**
    a. If you are using the Hamamatsu camera flip the switch shown in the image below. You will hear the fans of the camera start when you turn the camera on.
    b. The camera USB should always be plugged into a blue "USB 3.0" port like the one on the back of the computer as shown in the image below to the right.

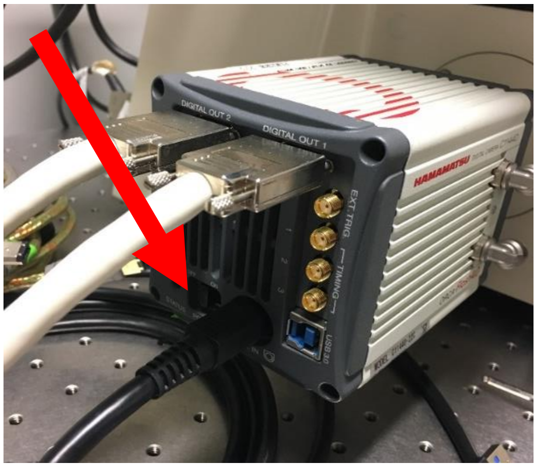

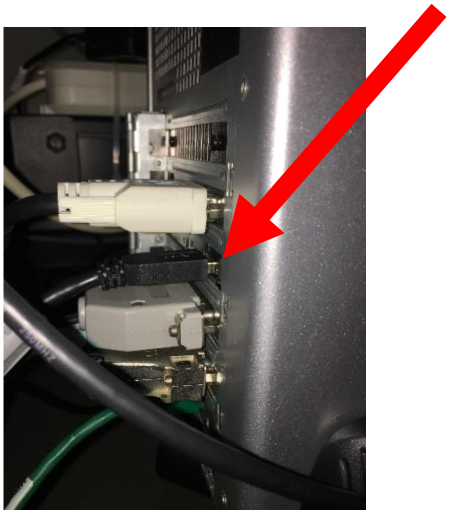

6. **Turn on Pressure Sensor**
    a. Hold the ON/OFF button to turn on the pressure sensor (displayed in PSI) indicated by the red arrow in the image to the right.

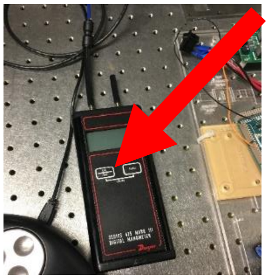

7. **Verify Arduino USB Connection is plugged in**
    a. The Arduino is indicated by the blue arrow in the image below. Verify the device is connected to the computer via USB. Small LEDS on the Arduino will light up when the Arduino is connected

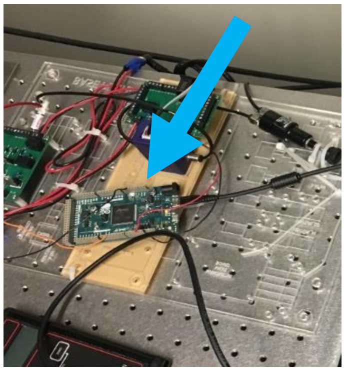

V. Running Experiment

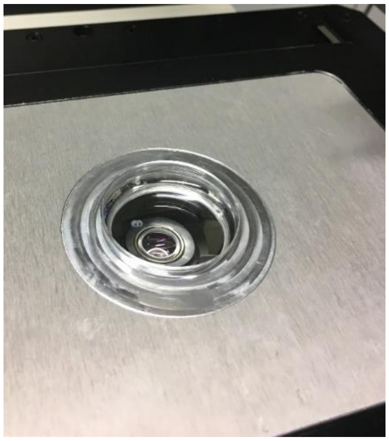

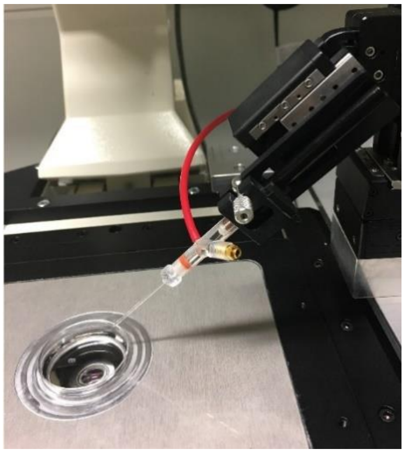

1. Place sample in sample holder on the microscope stage as shown in the left image above.

2. Load pipette with injection medium and mount onto manipulator as shown in the top right image above. Notice how the end of the pipette holder aligns with the end of the sliding stage and is parallel to the axis. Make sure the screw is tight to ensure a stable pipette

3. Load the application by clicking the file “launchapp.py” in the main folder downloaded from github. See github/section III for in depth software description. First a small black screen will appear followed by the GUI application. This may take up to 30s to load software the first time so be patient. The pop up screen below will show up (left image). Select the options shown in the right image (Camera = Hamamatsu Orca DCAM, com = com5, Res test = off) and click “save and exit”. You may need to select a different com port based on your computer (see trouble shooting/github to figure out which com port is appropriate).

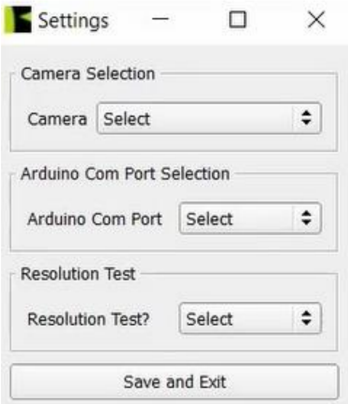

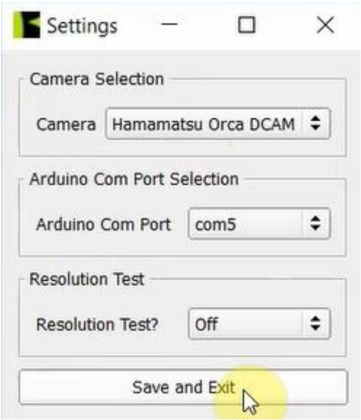

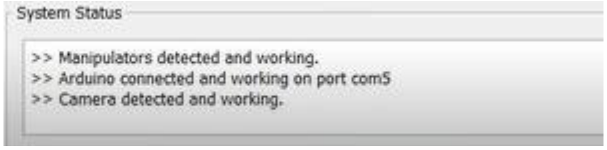

4. If the manipulator was loaded correctly you should lines in the system status shown in the image above, and numbers appear in the top right manipulator panel as shown in the top portion of the image to the right. Before submerging the pipette into the solution, it is necessary create outward pressure to prevent unwanted clogging. Slide the compensation pressure to an arbitrary value indicated by red arrow in image to right (24-45% works) and click set values. This will apply pressure to the pipette (you do not have to enter the other parameters yet although they are shown in this image).

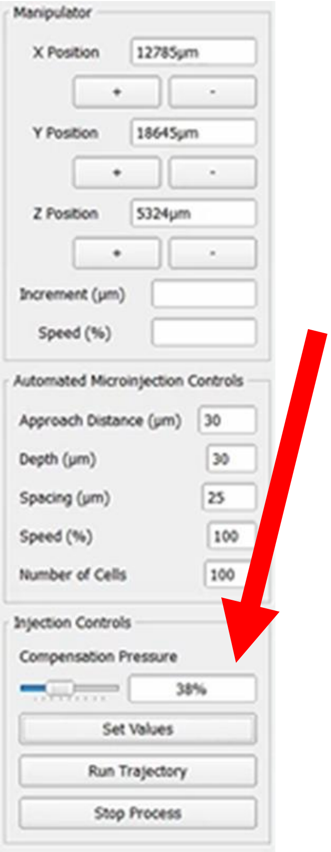

5. To obtain desired pressure turn the mechanical knob shown in the image below (clockwise to increase, counter clockwise to decrease). The units are in pound per square inch (1.08 PSI = 75mbar, which is what we use in our experiments for dye, 1.81PSI = 125 mbar for use with mRNA). This can vary widely based on the solution so it is more of a relative value and whatever pressure produces appropriate fluorescence is what you should use.

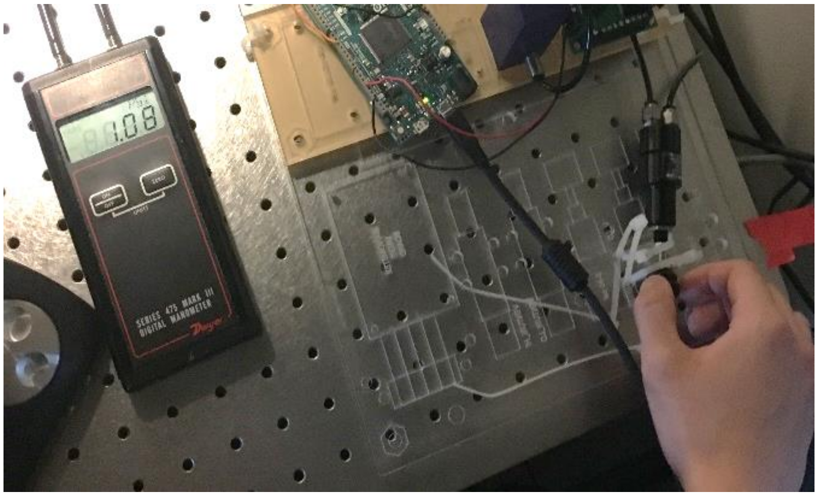

6. Bring the desired area into focus under the 10x objective and lower the pipette into the solution in this area. You can see that the pipette has been submerged by observing a slight dimple in the water as shown in the image below. Before going lower, search the entire Z area for the pipette using the microscope focus. Once you have found the pipette lower it into the solution in small steps and refocus. Repeat this process until the pipette is in the same focal plane as the tissue.

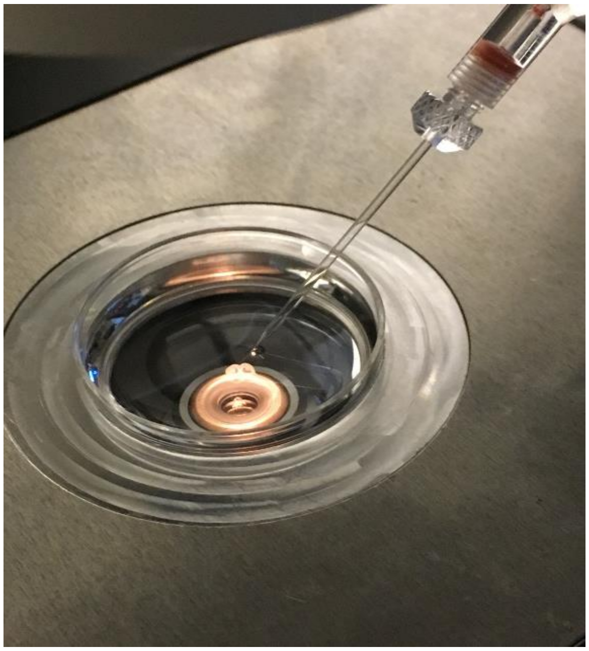

7. Depending on your microscope, switch the optical output from the microscope to the computer press the middle of the three buttons in the image to the right (shown with the caption "left/right" on the microscope). Watch the interface to verify. You should see something similar to the image below. You can adjust the exposure of the image by sliding the exposure bar in the GUI (not shown in image below, see image on page 4 under display settings).

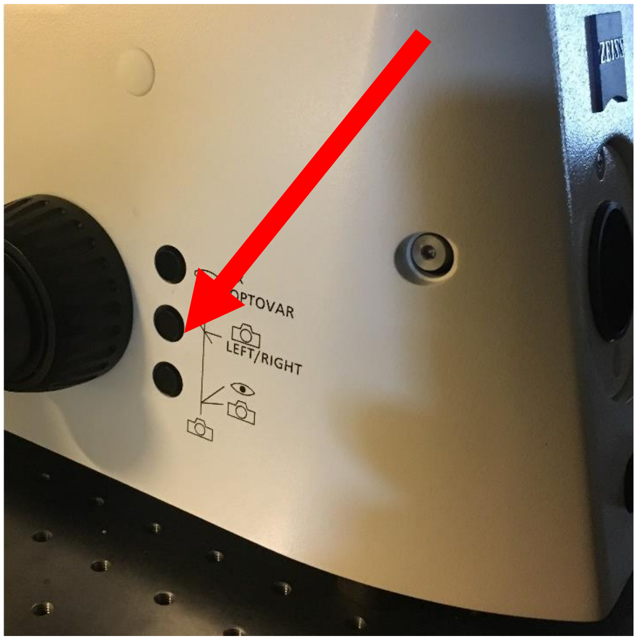

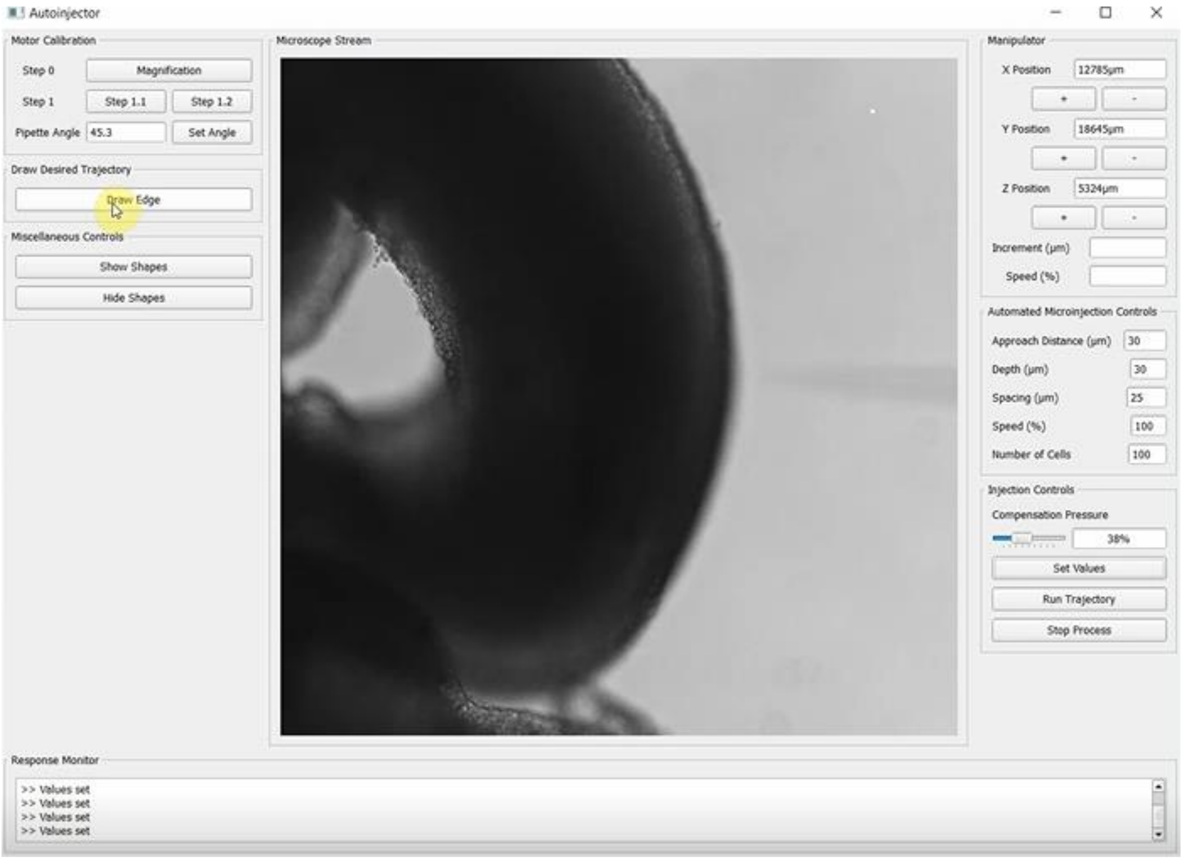

8. You may have to adjust the stage to get sample back to desired location.

9. **Calibration** – (see Movie EV1, and methods for more detail). Click the magnification button in the top left of the interface. A window will prompt you to select the magnification. Select 10x and press ‘Ok’.

10. Now, we need to calibrate the Autoinjector relative to the camera axes. Refocus the pipette tip and click the pipette tip with your cursor (number 1 in the image below). A white dot will appear where you clicked. Now, press step 1.1 as shown in the image below (number 2) and press OK in the popup window. The pipette will move in the Y direction.

11. Click the tip of the pipette again (number 3) and press step 1.2 as shown in the image below (number 4). The autoinjector is now calibrated.

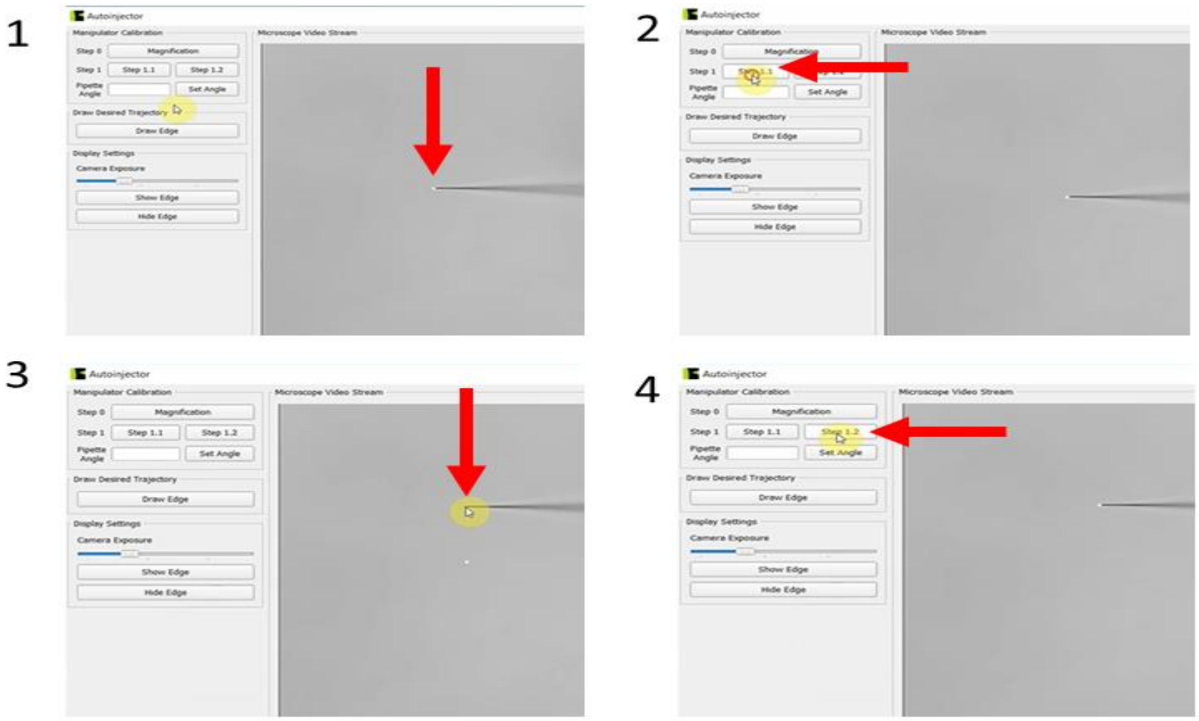

12. Now we need to enter the angle between the pipette X axis and the true x axis. This value should not change during operation of the device (it is usually 45.2 – 45.4). Thus, you do not need to check it every time. Simply enter “45.3” into the area and press “set angle”. It makes sense to check this value once per week to make sure it is not changing. To find this angle follow the following steps using the manipulator interface:

1 2 3

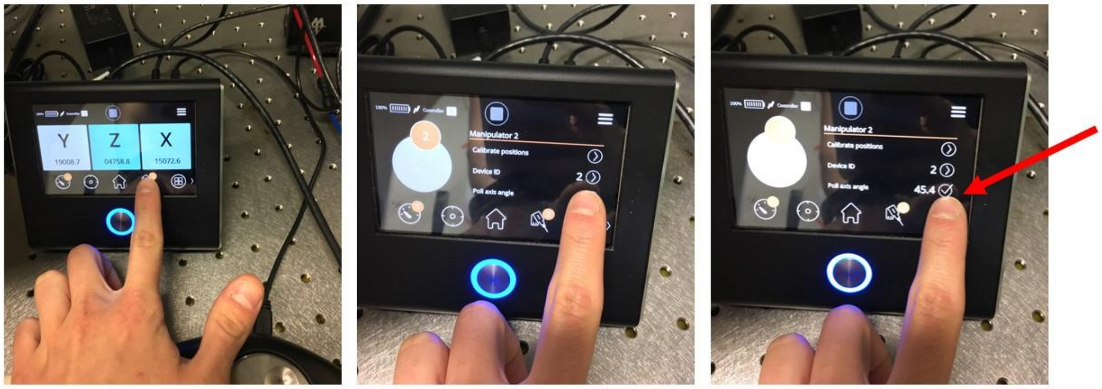

13. If desired, you may test that the calibration has worked well click the “Draw Edge” button as shown in the image to the right.

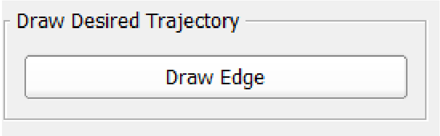

14. A popup screen will appear. Click and drag your cursor to trace a line as shown in the image below in black, a white line will appear where you have drawn the line in the interface. An error will appear if you draw the line towards the top of the image and then to the bottom of the image. Try to redraw the line if this occurs and make sure the line only goes in one direciton.

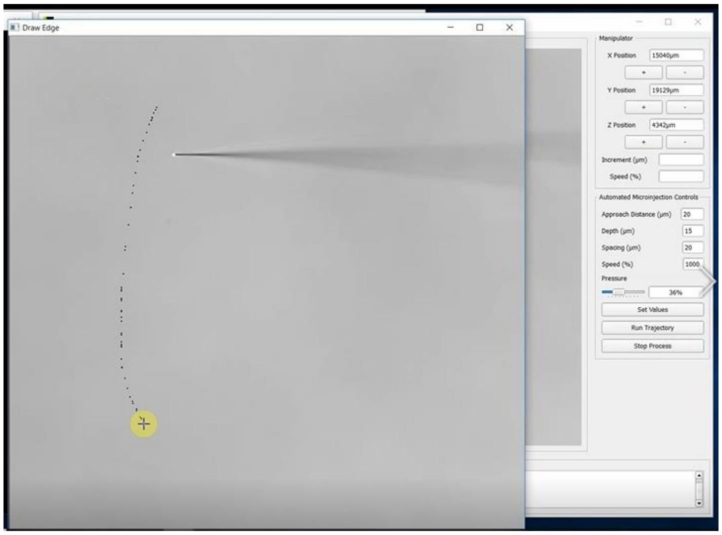

15. Bring the pipette close to the top of the line as shown in the image to the right and click the tip of the pipette.

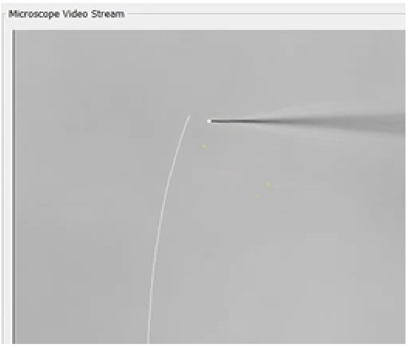

16. Enter desired parameters into the ‘Automated microinjection controls’ panel. The approach distance is the distance the pipette pulls out of the tissue before moving to the next injection distance (we use 20 – 40 µm), depth is depth into tissue depending on what cells you are targeting (10-15 for apical progenitors, 30-40 for neurons on basal side), spacing is distance along line between sequential injections (10 -30 µm depending on what you are targeting), speed is the speed of the pipette in µm/s (100 – 1000 is what we use).

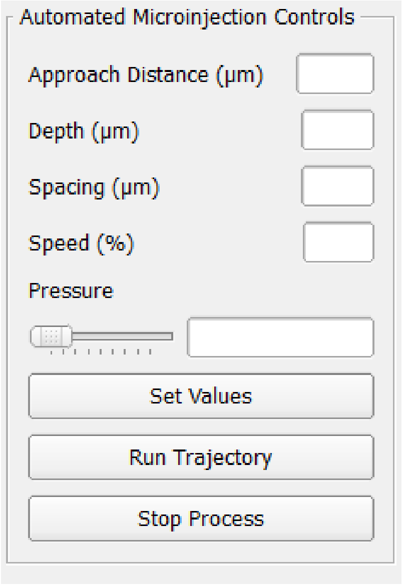

17. Click “Set values” button in the ‘Injection Controls’ panel on the bottom right of the interface.

18. Click “Run Trajectory” button this will start the trajectory. Observe the trajectory of the pipette, if this is satisfactory proceed, if it is not, recalibrate manipulators as described above, or see trouble shooting section. After the trajectory is finished, the number of attempts will be displayed in the bottom system status monitor.

19. Before injection, verify pipette is not clogged. Switch the viewing back to the microscope (see step 8), switch on epi shutter (beneath transhutter from step 6), and flip filter wheel (beneath objectives) to appropiate wavelength. You should see a small cloud of dye being emitted through the tip of the pipette. If you see no cloud, increase the pressure (step 5). If you increase the pressure (above 10 PSI) and see no cloud, the pipette is clogged (see section VI. Trouble shooting).

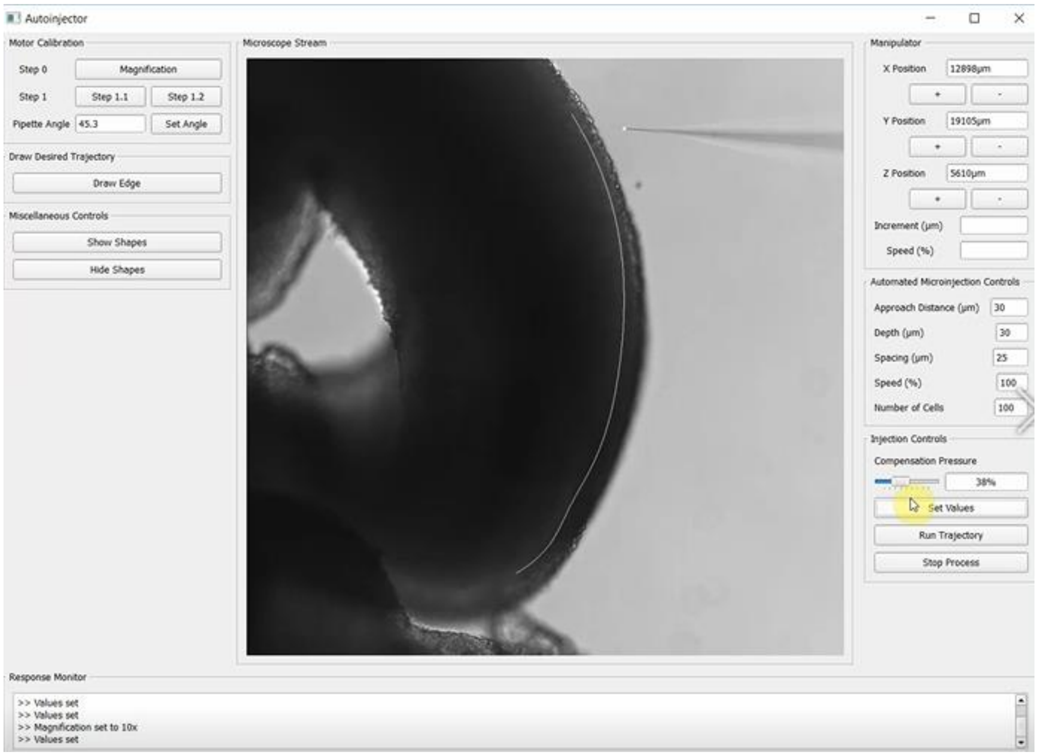

20. At this point, you may need to reposition the tissue and search for an appropiate focal plane for injection. The ideal tissue area will have an edge that is sharp within the same focal plane.

21. When the ideal focal plane is found adjust the pipette and redraw the desired trajectory by clicking the “draw edge” button, bring the pipette close to the top of the trajectory, and click the tip of the pipette as shown in the image above.

22. Click “Run Trajectory”

23. If at any point you wish to stop the process, click “Stop Process” which is located beneath the “Run Trajectory” button. The ideal slice will have 1 - 4 focal planes for injection and you can repeat step 20 – 22.

24. After use, pull pipette away from the slice, remove slice, and reposition stage to next slice if applicable. You do not need to recalibrate the pipette unless you change the pipette. Repeat steps 20 – 23 if desired. IMPORTANT, do note pull the pipette out of the solution or it will become clogged.

25. After you have completed injections, remove the pipette from the solution, remove slices, and turn off all devices (order is not important).

VI. Trouble Shooting

See the video tutorials for information on how to troubleshoot specific issues. The following issues are easily solvable. However, if your issue persists or is not on this list feel free to contact us.

- **Issue** – Manipulator does not load, pop up messages report errors.
  Causes -
  - The manipulator is not plugged into the back of the computer via Ethernet
  - the manipulator is not turned on
  - you have launched two instances of the application (two windows are open).
  - If you turn on the computer and try to run the application right away the manipulator will not be detected, it needs about 5 minutes after turning on to properly load.
- **Solution** - Verify connections and turn on manipulator (section III), or close programs and wait 2 minutes to verify programs are shut. Relaunch the application.

- **Issue –** When running trajectory, pipette goes to first location but freezes in place.
  Cause - There is an Arduino connection problem. Confirm this by minimizing the interface and looking at the black box window behind the interface. If you see the words "there is most likely an Arduino error" then there is most likely an Arduino error.
  Solution – Unplug the Arduino USB and replug the USB, if problem persists try another USB port.

- **Issue** - Camera feed is not displayed, but is replaced with text that says "CAMERA ERROR".
  Causes – The camera is not turned on, the camera is not properly connected, there is another instance of the application open (two windows of the same app). The wrong software is implemented for the wrong camera.
  Solution – Verify proper connections are made and device is on, close applications and wait 2 minutes for applications to be killed properly, make sure correct camera was selected from the dropdown menu.

- **Issue** - Pipette is clogged
  Causes - molecules aggregate, accidently punctured tissue, pipette is not ideal shape.
  Solutions – centrifuge injection solution and remove only supernatant, replace pipette, pull pipette at 2 deg lower, and lower pull value to 50.
  Elena and Christiane can provide good feedback on this as well.

VI. Movie Tutorial Descriptions

1. **“Movie EV4.mp4”** – This video shows you how to start the program, how to calibrate the autoinjector, and how to run the Autoinjector.

2. **“Movie EV5.mp4”** – This video shows you the errors that come up when there is a hardware problem such as parts not being plugged in. The video suggests steps to take to fix these problems.

3. **“Movie EV6.mp4”** – This video displays an error that comes up sometimes when drawing the line. If you have this error, this video tells you how to fix this problem.

4. **“Movie EV7.mp4”** – This video shows you where in the GUI to see the number of injections that were completed.

VII. Guidelines

These guidelines provide user with practical notes for implementing automated microinjection. We limit our discussion to microinjection into single neural stem cells and neurons in tissue. Further optimization may be needed when users are attempting to adapt the Autoinjector for microinjecting other types of tissue or cell types.

Table 1 – Summary of parameters that affect microinjection efficiency

Parameter	Description	Typical Values	Troubleshooting
Pipette Shape	The shape of the pipette is controlled by the pipette puller parameters and plays a role in minimizing tissue damage and pipette clogging.	- See figure 1 for a picture of a good injection pipette.<br>- Microinjection pipettes have a long taper<br>- The tip of the pipette is typically < 0.5µm.	- If pipette is clogged, decrease temperature by 1°C to create larger tip opening.<br>- Refer to previous work for further guidance [1].
Chemical Nature (ability to aggregate)	The viscosity of the solution is controlled by the magnitude of molecule aggregation. If solution is too viscous it will clog. This is the most common problem encountered during microinjection.	- Dyes are usually less viscous and do not clog the pipette as easily.<br>- Proteins and mRNA(s) aggregate more readily and are thus more viscous and clog the pipette frequently.	- Centrifuge solution before injecting and every 30 minutes following.<br>- Optimize buffer compositio.<br>- Consider using lower concentration if possible, or lower number of unique molecules if injecting multiple mRNA(s)/protein.<br>- Once pipette is in solution, do not remove it out of the solution. This furthers potential for aggregation.
Injection Pressure	The injection pressure controls the volume of liquid injected, mechanical stress to cells, and	- When injecting dye we use a pressure of 75 mBar (1.08 PSI).<br>- When injecting proteins/mRNA we typically use	- If the pipette is clogged, move the pipette away from the tissue slice and temporarily increase pressure to 1000 mBar.<br>- If solution is very viscous, increase pressure up to 250

	pipette clogging.	a pressure of 125 mBar (1.81 PSI).	mBar.
Spacing between injections	Spacing between injections will affect the ability to resolve individual injected cells, if the spacing is too close it is difficult to differentiate processes. It will also affect mechanical stress on the slice with lower spacing inducing more stress which may affect viability.	- For APs we use a spacing > 15 µm. If we want to target large numbers of cells within the same slice we will use 15 µm.<br>- For neurons on the basal surface we used a larger spacing (30-400 µm) to be able to resolve neurites.	- Consider the cells being targeted, their morphology and if they have processes. If they do, increase spacing.<br>- If the cells are particularly sensitive to stress and the experiment requires culturing the cells for several days post injection, minimizing space between injections may increase efficiency.
Depth of injection	The depth of microinjection should be minimized to prevent cell damage but maximized to increase injection yield.	- 10 µm for apical progenitors (APs).<br>- 25 µm for neurons from basal surface.	- It is helpful to vary injection depth and quantify yield as we did in Figure 3 of the main manuscript (varied from 10 – 25 µm for APs).
mRNA translation efficiency	The efficiency of injected mRNA translation may vary based on cell type targete and mRNA type.	- 25% success in hindbrain mouse organotypic slices[2].<br>- ~45% success in organotypic mouse telencephalon[3,4].	- Troubleshooting injection parameters listed above will help deliver consistent mRNA concentrations to cells.<br>- It may be helpful to inject different concentrations of mRNA and quantify efficiency of translation. This can be done with a slice in an experiment and take ~20 minutes to inject.

PREPARING FOR MICROINJECTION

How to obtain a good pipette

Pipettes for microinjection into single neural stem cell (or neurons) in tissue typically have a long taper and a tip opening of < 0.5µm. A detailed description and a caption of what we consider to be a good pipette can be found in previous work [1]. We routinely use a P-97 Flaming Brown pipette puller to pull our pipettes. The glass capillaries are from Sutter Instruments (0.9 ID x 1.2 OD).

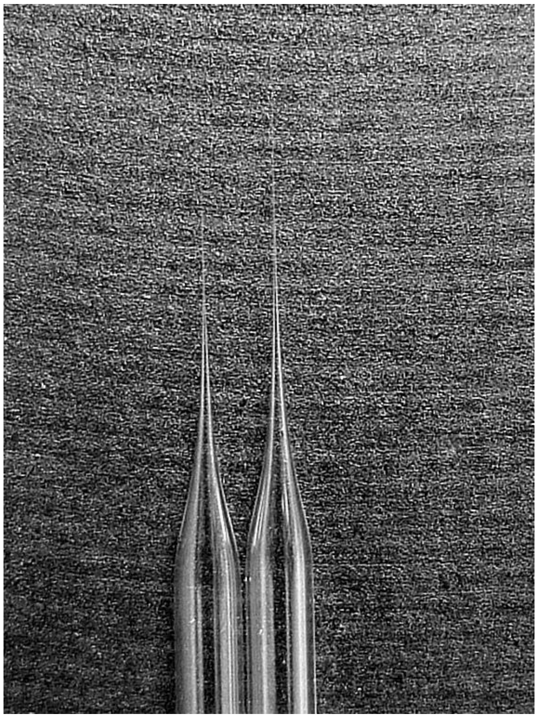

Figure 1: Pipette shape. An example of a good and a bad pipette is shown (image acquired and processed by Felipe Mora-Bermúdez)

The pipettes are better pulled right before use and then kept in a dish or box to protect the pipettes from dust. Storing the pipette long time after they are pulled (> 2 days) is not recommended.

As for the parameters of the puller, one should keep in mind that the puller parameters (Time, heat, Velocity, etc) are strongly depending on the puller itself and on the specific ramp test temperature of the pipette one uses. The ramp test must be determined by the user before starting to use the puller. We provide as an example the parameters that we did use for our pipettes, and the corresponding ramp test of the glass capillaries that were used.

The chemical nature of the microinjection solution and its effects on the efficiency of injection

Microinjection relies on the use of pressure to introduce chemicals into cells, as such, one can microinject virtually every compound (and their combination), irrespective of its chemical nature. The success of microinjection can be influenced by the chemical nature of the compound to be microinjected. In particular, the intrinsic tendency of a compound

to form aggregates is going to increase the likelihood of pipette clogging, thus decreasing the efficiency of microinjection.

For example, microinjection dyes (Dextran-conjugated fluorescent dyes; Lucifer Yellow, uncoupled Alexa dyes, etc.) show little tendency to aggregate, while RNAs and proteins show a higher tendency to aggregate. To mitigate the tendency of RNA to aggregate, we recommend denaturing the RNA by boiling it at 92C for 45-60 seconds. To mitigate the tendency of proteins to aggregate, one should choose a solution that prevents protein aggregation and maintains protein solubility and stability. It is not possible to give a one-solution-fits-all recommendation in these cases, so every researcher should take care of finding the correct working conditions.

MICROINJECTION

Determining inter-injection spacing

While setting the spacing parameter, one should consider two factors:

1. Tissue Viability:

Choosing a small spacing (< 10 µm) might have detrimental effects on tissue (and cell) viability due to the increased mechanical stress. In our hands, a minimum of 15 µm spacing was used. This (or larger) spacing was found not to have any noticeable effect on tissue viability and tissue structure. We do not recommend using a smaller spacing. In case the user needs to use a smaller spacing then we do recommend running viability controls to rule out detrimental effects.

2. Ability to discriminate single cells post injection: A small spacing (< 10 µm) is going to negatively affect single cell resolution, as cells will be too close to be distinguished unambiguously. We recommend using > 15-20 µm to secure good single cell resolution in case one wants to inject apical progenitors (APs). For injecting neurons, we recommend using > 30 µm, as the neuron structure is normally quite complex and extends on a very large area.

EFFICIENCY OF TRANSLATION FOR MRNA MICROINJECTION

The efficiency of translation for mRNA microinjection was found to range from c.ca 25% in the hindbrain[2] to c.ca 45% in the telencephalon[3,4] (and present work). The low efficiency of translation might appear to be a limitation at a first glance. However, it provides as a good internal control, as the user can score separately the RFP-positive cells and the RFP-negative cells. If the phenotype depends on the expression of the exogenous mRNA(s), then one expects the phenotype to be present in RFP-positive cells and absent in the RFP-negative cells. The user should keep this in mind while planning the experiment and the appropriate controls.

1. Wong, F. K., Haffner, C., Huttner, W. B. & Taverna, E. Microinjection of membrane-impermeable molecules into single neural stem cells in brain tissue. *Nat. Protoc.* **9,** 1170–1182 (2014).

2. Taverna, E., Haffner, C., Pepperkok, R. & Huttner, W. B. A new approach to manipulate the fate of single neural stem cells in tissue. *Nat. Neurosci.* **15,** 329–337 (2012).

3. Florio, M. *et al.* Human-specific gene ARHGAP11B promotes basal progenitor amplification and neocortex expansion. *Science (80-.).* **347,** 1465–1470 (2015).

4. Tavano, S. *et al.* Insm1 Induces Neural Progenitor Delamination in Developing Neocortex via Downregulation of the Adherens Junction Belt-Specific Protein Plekha7. *Neuron* **97,** 1299–1314.e8 (2018).
